# Integrated genomics-based mapping reveals the genetics underlying maize flavonoid biosynthesis

**DOI:** 10.1186/s12870-017-0972-z

**Published:** 2017-01-18

**Authors:** Min Jin, Xuehai Zhang, Mingchao Zhao, Min Deng, Yuanhao Du, Yang Zhou, Shouchuang Wang, Takayuki Tohge, Alisdair R. Fernie, Lothar Willmitzer, Yariv Brotman, Jianbing Yan, Weiwei Wen

**Affiliations:** 1 National Key Laboratory of Crop Genetic Improvement, Huazhong Agricultural University, Wuhan, 430070 China; 2 Max Planck Institute for Molecular Plant Physiology, 14476 Potsdam-Golm, Germany; 3 Department of Life Sciences, Ben Gurion University of the Negev, Beersheva, Israel; 4 Key Laboratory of Horticultural Plant Biology (Ministry of Education), Huazhong Agricultural University, Wuhan, 430070 China

**Keywords:** Maize, Flavonoid, Natural variation, Linkage mapping, Association analysis, Co-expression network

## Abstract

**Background:**

Flavonoids constitute a diverse class of secondary metabolites which exhibit potent bioactivities for human health and have been indicated to play an important role in plant development and defense. However, accumulation and variation of flavonoid content in diverse maize lines and the genes responsible for their biosynthesis in this important crop remain largely unknown. In this study, we combine genetic mapping, metabolite profiling and gene regulatory network analysis to further enhance understanding of the maize flavonoid pathway.

**Results:**

We repeatedly detected 25 QTL corresponding to 23 distinct flavonoids across different environments or populations. In addition, a total of 39 genes were revealed both by an expression based network analysis and genetic mapping. Finally, the function of three candidate genes, including two UDP-glycosyltransferases (*UGT*) and an oxygenase which belongs to the flavone synthase super family, was revealed via preliminary molecular functional characterization.

**Conclusion:**

We explored the genetic influences on the flavonoid biosynthesis based on integrating the genomic, transcriptomic and metabolomic information which provided a rich source of potential candidate genes. The integrated genomics based genetic mapping strategy is highly efficient for defining the complexity of functional genetic variants and their respective regulatory networks as well as in helping to select candidate genes and allelic variance before embarking on laborious transgenic validations.

**Electronic supplementary material:**

The online version of this article (doi:10.1186/s12870-017-0972-z) contains supplementary material, which is available to authorized users.

## Background

Maize (*Zea mays L*.) is the world’s most widely grown crop for food, animal feed, biofuel and other industrial materials, and displays the highest global grain production [1]. By 2050, it is estimated that the human population will reach 9 billion [2]. Increasing yield while providing added nutritional value in maize is thus imperative to meet the growing nutritional demand of the huge global population [3, 4].

Flavonoids are a class of phytochemicals containing a C6-C3-C6 carbon framework. Based on the oxidation and saturation in the heterocyclic ring, flavonoids can be classified into six subclasses namely flavone, flavonol, flavanone, flavanol, anthocyanin and isoflavone [5]. Flavonoids have potent anti-inflammatory and anti-carcinogenic activities and they may thus offer protection against major diseases such as cardiovascular diseases, coronary heart diseases and tumor [6, 7]. In plants, the major states of flavonoids are modified with sugar or hydroxyl moities to facilitate stable storage and confer various biological functions. Moreover, the different colors exhibited by flavonoids can attract pollinators and thus are highly important in plant reproduction [8, 9]. They can additionally function as an UV absorbing compounds protecting plants from the UV-B radiation [10, 11], and can also serve as anti-pathogen such as maysin and GCA (chlorogenic acid) in maize [12, 13]. Flavonoids are necessary for conditional male fertility in maize [14] and are also involved in seed coat development [15], regulating the transport of phytohormones [16, 17], and providing signals to symbionts in plants [18]. Therefore, understanding flavonoid biosynthesis in maize and the genetic basis underlying natural variation of the contents of members of this compound class is essential for maize enhancement in terms both of improving its nutritional value and in maintaining yields by ensuring stress tolerance.

Flavonoid biosynthesis is one of the most intensively studied areas in plant secondary metabolism, and synthesis of most flavonoids have the same steps which are highly evolutionarily conserved [5, 19]. The synthesis starts with the formation of naringenin chalcone by chalcone synthase (CHS) encoded in maize by locus *C2* (*colorless 2*) [20]. The product naringenin chalcone is then converted by chalcone isomerase (CHI) to generate the flavanone naringenin, which serves as a key precursor for various flavonoids [21]. Through the action of flavanone-3′-hydroxylase enzyme (at the *Pr1* locus), naringenin can then be converted into another flavanone, eriodictyol [22, 23]. The biosynthesis of flavones begins with flavanones. Flavones are often *O*- or *C*- glycosylated by glycosyl transferases. There are two major different classes of flavone synthase (FNS), FNSI and FNSII. FNSI type enzymes are soluble Fe^2+^ oxoglutarate-dependent dioxygenases (2-ODDs) and the maize FNSI-1 shows similar enzymatic activity with PcFNSI [24] and produces flavone; while FNSII type enzymes are oxygen- and NADPH-dependent cytochrome P450 membrane-bound monooxygenases [25]. Generally, the FNS protein can prepare aglycone backbones prior to final *O*-linked modifications [26]. On the other hand, the well characterized F2H (flavanone 2-hydroxlase) can initiate the hydroxylation of flavanones to the 2-hydroxyflavanones. 2-hydroxyflavanones then serves as the substrate [27] for *C*-glucosylation to form various flavones 6-*C*- or 8-*C*-glucosides [28] with the accompany of *C*-glycosyltransferase (CGT) [29–32]. The maize *F2H* (*CYP93G5*) was identified through a genome wide expression and ChIP analysis, and co-expression of *ZmF2H1* and *UGT708A6* results in the formation of flavone *C*-glycosides [29, 31].

Besides the synthetic enzymes involved in the pathway, considerable regulation by transcription factors (TFs) has been elucidated. The much studied *Pericarp1* (*P1*) is an R2R3-MYB transcription factor which can control the accumulation of various phenylpropanoids by activating a subset of flavonoid biosynthetic genes [33, 34]. The *C1* like R2R3-MYB and *R1* like bHLH interacting factors also activate the pathway [35]. Flavonoid synthesis is completed in the cytoplasm; however, the final location of different flavonoids is diverse and as such transport processes are necessary for flavonoid movement within and between cells. Three kinds of transporters namely proton-dependent transporters [36], ATP binding cassette-type transporters [37] and MATE-type transporters [38] may be involved in these transport processes. Natural variations in these TFs and enzymes contribute to the divergence of flavonoid accumulation. Many genes in the flavonoid pathway have been cloned using a combination of classical genetics and biochemistry, however recently transposon based mutagenesis and T-DNA tagging approaches have also been employed. In maize, dozens of genes involving in flavonoid biosynthesis were identified based on linkage analysis, transposon tagging approaches and homology-based cloning [22, 39], however, our understanding of the genetic control of the biosynthetic pathways underlying maize flavonoids biosynthesis remains fragmentary [19].

In recent years, the rapid development of metabolomics and adoption of diverse populations for genetic mapping has provided us with unprecedented knowledge concerning the regulation of the abundance of the diverse chemical components in plants [40]. With the aid of high-throughput genotyping and metabolomics data, metabolic QTL were identified in a rice Zhenshan 97 and Minghui 63 recombinant inbred line (RIL) population, and some of the candidate genes for flavonoid content were further validated by examining over-expression transgenic rice lines [41]. A novel gene (*BETA GLUCOSIDASE 6, BGLU6*) was recently identified to be responsible for the production of flavonol 3-*O*-gentiobioside 7-*O*-rhamnoside (F3GG7R) in an Arabidopsis RIL population [42, 43]. In maize, hundreds of loci associated with metabolites from multiple pathways including flavonoid metabolism were identified, following genome wide association studies (GWAS) on a diverse maize population that revealing the genetic influences underlying metabolic variation [44]. In addition, near isogenic lines (NILs) containing *P1-rr* and *P1-ww* were used to study the co-expression and direct target genes of the R2R3-MYB transcription factor *P1* [31]. Since *P1* was proven to regulate some well-known genes involved in flavonoid biosynthesis, such as *FLS1* and *A1* through targeted molecular experiments [45], this study represented a great advance to systematically comprehend its gene regulatory circuitry. Maysin (*C*-glycosyl flavone) present in maize silks confers natural resistance to the corn earworm (*Helicoverpa zea*), which can cause severe damages on maize in the Americas [12]. Two loci that are capable of conferring salmon silks phenotypes, *salmon silks 1* (*sm1*) and *salmon silks 2* (*sm2*) were identified through QTL mapping [30] in 2004. And previous genetic analyses predicted *P1* to be epistatic to the salmon silk mutation [13]. Based on the available *sm1* and *sm2* mapping information and knowledge of the genes regulated by *P1* [13, 31], the molecular identification of the *sm1* and *sm2* gene products are revealed as a UDP-rhamnose synthase and a rhamnosyl transferase, respectively [12]. The molecular characterization of *sm1* and *sm2* therefore completes the maysin biosynthetic pathway. It can thus be anticipated that deep probing of further profiling studies will facilitate the elucidation of the genetic complexity of maize flavonoid biosynthesis. Indeed, integrative approaches are increasingly applied to enhance our understanding of metabolic pathway structure and regulation and how these affect the end-phenotypes of plants [46].

Previously, comprehensive metabolic profiling using liquid chromatography tandem mass spectrometry (LC-MS/MS) was carried out in mature maize kernels coming from several populations. Combined linkage analysis and GWAS was carried out on the resultant datasets which led to the identification of a variety of loci involved in multiple biosynthetic pathways [44, 47]. Taking advantage of the informative dataset generated from these previous studies, we here combine genetic mapping, metabolite profiling and gene regulatory network analysis to further enhance understanding of the maize flavonoid pathway. To this end the function of three candidate genes, including two maize UDP-glycosyltransferases (*UGT*) and an oxygenase which belongs to the flavone synthase super family, was revealed through preliminary molecular functional characterizations including re-sequencing to access allelic variance and candidate gene association as well as reverse genetic experiments employing transgenic approaches. We discuss the obtained results not only in the context of our understanding of maize flavonoid biosynthesis but also in the context of maize genetic improvement both from the perspective of its nutritional content and in terms of its ability to withstand biotic and abiotic stress.

## Results

### Variation of flavonoids in different maize populations

**Fig. 1:**
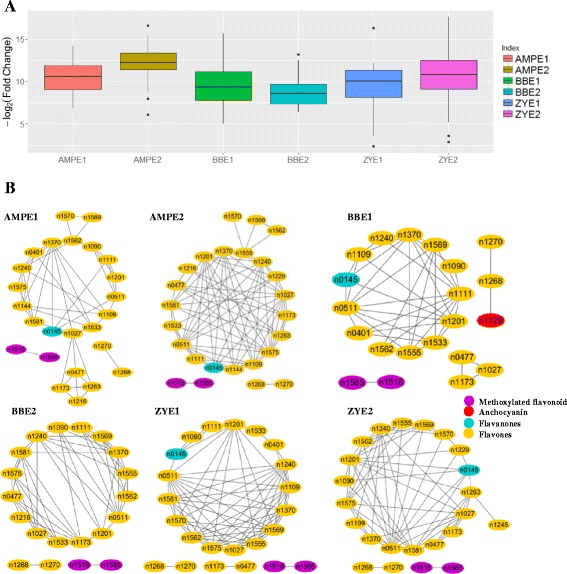
Distribution of log_2_-fold changes and correlation coefficient based network of all flavonoids measured in AMP and two RIL populations. **a** Box plots showing the log_2_ value of fold changes of 29 flavonoids among the AMP and both BB and ZY RILs. Data from different environments (experiments) for AMP and each RIL population are shown. **b** Correlation coefficient based network of all flavonoids in each experiment for AMP and both BB and ZY populations. *R* ≥ 0.3 for correlation coefficient between two flavonoids was used to construct the network

An association mapping panel (AMP) and two RIL populations were planted in multiple environments (simply called AMPE1, AMPE2 for AMP, BBE1, BBE2 for BB RIL population, and ZYE1 and ZYE2 for ZY RIL population, which were described in detail in “Materials and Methods”) and the mature kernels harvested from these six field experiments were used for LC-MS/MS based metabolite profiling. In our previous metabolome-based GWAS study, 983 metabolite features were identified in the AMP [44]. 184 of these 983 metabolite features with chemical or putative annotations were analyzed in BB and ZY RIL populations subsequently [47]. In this study, we extract the profile of flavonoids from these previous datasets, which includes 29 flavonoids and five of them were chemically annotated. Briefly, these 29 flavonoids can be classified into flavones, flavanones, anthocyanins and methoxylated flavonoid. Among them, 28, 27, 23, 22, 25, 24 flavonoids were found in AMPE1, AMPE2, BBE1, BBE2, ZYE1, ZYE2, respectively, 15 flavonoids were detected in all the six environments (Table 1). The AMP and both RIL populations manifested great diversity in their flavonoid levels (Additional files 1 and 2: Tables S1 and S2), as indicated by the distribution of the log_2_ value of fold changes (Fig. 1a). In AMP, all flavonoids have broad-sense heritability (H^2^) greater than 0.3 and over 65% of flavonoids have H^2^ greater than 0.7. Over 45% and 60% of flavonoids have H^2^ greater than 0.5 in BB and ZY populations, respectively (Additional file 3: Figure S1). Correlation coefficient networks were also constructed based on flavonoid levels detected in each experiment, respectively, which demonstrated a clear separation between methoxylated flavonoids and other flavonoids, and most flavones were consistently linked to each other with R > 0.3 (Fig. 1b).

**Table 1:** Detailed information of 29 flavonoids detected in this study

No.	Peak no.	Level^a^	Ret. Time (min)	Putative flavonoid name	Flavonoid Class	Mol formula	AMPE1	AMPE2	BBE1	BBE2	ZYE1	ZYE2
1	n1129	A	4.7	Cyanidin 3-*O*-glucoside	Anthocyanin	C21H21O11	√	√	√	×	√	√
2	n1518*	C	6.11	Methoxylated flavonoid 3-*O*-hexoside	Methoxylated flavonoid	C23H24O12	√	√	√	√	√	√
3	n1585*	B	4.94	Methoxylated flavonoid di-*O*-hexoside	Methoxylated flavonoid	C29H34O17	√	√	√	√	√	√
4	n1109	A	8.68	Apigenin 7-*O*-glucoside	Flavones	C21H20O10	√	√	√	×	√	√
5	n0511*	C	6.96	Apigenin *C*-hexosyl-*C*-pentoside	Flavones	C26H28O14	√	√	√	√	√	√
6	n1201*	C	7.53	Apigenin *C*-pentosyl-*C*-pentoside	Flavones	C25H26O13	√	√	√	√	√	√
7	n1268*	C	8.49	Apigenin *C*-pentosyl-*O*-coumaroylhexoside	Flavones	C35H34O16	√	√	√	√	√	√
8	n1555	C	7.16	Apigenin di-*C*-hexoside	Flavones	C27H30O15	×	√	√	√	√	√
9	n1090	C	8.92	Apigenin-*C*-pentoside	Flavones	C20H18O9	√	×	√	√	√	√
10	n1270*	B	7.81	*C*-pentosyl-apigenin *O*-caffeoylhexoside	Flavones	C35H34O17	√	√	√	√	√	√
11	n1370*	A	11.8	Chrysoeriol	Flavones	C16H12O6	√	√	√	√	√	√
12	n1229	C	8.11	Chrysoeriol *C*-hexosyl-*O*-pentoside	Flavones	C27H30O15	√	√	×	×	×	√
13	n1562*	B	7.74	Chrysoeriol *C*-hexosyl-*O*-rhamnoside	Flavones	C28H32O15	√	√	√	√	√	√
14	n1569*	C	6.63	Chrysoeriol di-*C*-hexoside	Flavones	C28H32O16	√	√	√	√	√	√
15	n1245*	C	8.18	Chrysoeriol di-*O*-hexoside	Flavones	C28H32O16	√	√	√	√	√	√
16	n1144	C	8.83	Chrysoeriol *O*-hexoside	Flavones	C22H22O11	√	√	×	×	×	×
17	n1240*	C	9.4	Chrysoeriol *O*-rhamnosyl-*O*-hexoside	Flavones	C28H32O15	√	√	√	√	√	√
18	n0401	C	9.15	Methylchrysoeriol *C*-hexoside	Flavones	C23H24O11	√	×	√	×	√	×
19	n1027*	B	11.7	Tricin	Flavones	C17H14O7	√	√	√	√	√	√
20	n1173*	C	11.5	Tricin 4′-*O*-(erythro-β-guaiacylglyceryl) ether	Flavones	C26H24O10	√	√	√	√	√	√
21	n0477*	C	11.9	Tricin 4′-*O*-(threo-β-guaiacylglyceryl) ether	Flavones	C27H26O11	√	√	√	√	√	√
22	n1263	C	8.8	Tricin 4′-*O*-(threo-β-guaiacylglyceryl) ether *O*-hexoside	Flavones	C33H36O16	√	√	×	×	×	√
23	n1216	B	13.2	Tricin derivative	Flavones		√	√	√	√	×	×
24	n1570	B	8.22	Tricin *O*-pentosyl-*O*-hexoside	Flavones	C28H32O16	√	√	×	×	√	√
25	n1575	C	9.35	Tricin *O*-rhamnosyl-*O*-hexoside	Flavones	C29H34O16	√	√	×	√	√	√
26	n1581	C	11.2	3′,4′,5′-Tricetin *O*-rhamnosyl-*O*-hexoside	Flavones	C30H36O16	√	√	×	√	√	√
27	n1533	C	9.07	3′,4′,5′-Tricetin-*O*-hexoside	Flavones	C24H26O12	√	√	√	√	√	×
28	n1111	A	7.75	Vitexin	Flavones	C21H20O10	√	√	√	√	√	×
29	n0145*	A	11.6	Naringenin	Flavanones	C15H12O5	√	√	√	√	√	√

^a^Identification level (A; B; C; D)- (A) standard or NMR; (B) MS/MS; (C) MSE; (D) MS only

*represents the flavonoid that was detected in all six environments

“√” and “ × ” indicated that the flavonoid was detected or not in each environment

### GWAS for flavonoid levels

A total of 79 loci were identified by GWAS at significance level of *P* ≤ 1.8 × 10^−6^ in two experiments (AMPE1, AMPE2) (Table 2). Briefly, 51 loci were identified for 23 flavonoids in AMPE1, with an R^2^ (explained phenotypic variation) ranging from 6.84 to 19.77% and a mean of 8.93%; while 28 loci were detected for 18 flavonoids in AMPE2. Each locus could explain phenotypic variation ranging from 6.88 to 19.48%, with a mean of 10.19% (Additional file 4: Table S3). Of the 17 common flavonoids for which significant loci were detected in both experiments, a total of 42 and 27 loci were detected in AMPE1 and AMPE2, respectively, and 12 of which were conserved for the same flavonoids in both experiments (Additional file 5: Figure S2A). The detailed information for GWAS results including P value and R^2^ of each locus, physical position and minor allele frequency (MAF) of lead SNP and the most likely candidate gene and its annotation are provided in Additional file 4: Table S3. All potential candidate genes and their functional annotations within 100 kb (50 kb upstream and downstream of the lead SNP) of the loci identified from GWAS are listed in Additional file 6: Table S4.

**Table 2:** Summary of significant loci-trait associations identified by GWAS and QTL identified by linkage mapping

	AMPE1^*^	AMPE2	BBE1	BBE2	ZYE1	ZYE2
Number of traits^a^	23/28	18/27	22/23	22/22	23/25	23/24
Number of loci^b^	51	28	51	55	64	59
Average loci per trait^c^	2.2 ± 1.7	1.6 ± 0.8	2.3 ± 1.1	2.5 ± 1.2	2.8 ± 1.0	2.6 ± 1.3

^*^AMPE1 and AMPE2 represent the two experiments conducted on the association panel; BBE1, BBE2 and ZYE1, ZYE2 represent the two experiments conducted on both BB and ZY populations; BB, linkage population B73/By804 RIL; ZY, linkage populationZong3/Yu87-1 RIL

^a^Number of traits having significantly associated loci or QTL (before slash), number of total detected traits (after slash)

^b^Number of significant loci detected in each experiment on the association panel (P ≤ 1.8 × 10^−6^, MLM) and QTL detected in each RIL population (LOD ≥ 2.5)

^c^Average number of significant loci (or QTL) detected per trait ± s.d

### Linkage mapping for flavonoid levels in the two RIL populations

For the BB population, 51 and 55 QTL were mapped for 22 flavonoids in BBE1 and BBE2, respectively (Table 2). A total of 99 QTL were detected for the 19 common flavonoids in both experiments (Additional file 7: Table S5), 12 QTL of which were conserved for the same flavonoid in both experiments (Additional file 5: Figure S2B). The percentage of phenotypic variation (R^2^) that each QTL could explain ranged from 2.94 to 76.79%, with a mean of 10.33% (Additional file 7: Table S5). Twenty-nine QTL that explained greater than 10% of the phenotypic variation (*R* ^2^ = 10.03-76.79%) were identified.

In the ZY population, a total of 123 QTL were detected in the two experiments (Table 2). Each QTL could explain between 2.85 and 23.17% of phenotypic variation, with an average variation of 9.35%. 47 QTL were identified that explained greater than 10% of the variation (*R* ^2^ = 10.02–23.17%). Specifically, 64 QTL were detected for 23 flavonoids in ZYE1 (Table 2), with an R^2^ range of 4.81 to 23.17% and a mean of 9.38%, while in ZYE2, 59 QTL were identified for 23 flavonoids (Table 2) and an R^2^ range of 2.85–18.34% with a mean of 9.31% (Additional file 7: Table S5). Of the 21 common flavonoids for which could detected QTL in both experiments, a total of 57 and 51 QTL were detected in ZYE1 and ZYE2, respectively, 27 of which were conserved for the same flavonoids in both experiments (Additional file 5: Figure S2C).

Linkage mapping results from both BB and ZY populations indicated that most flavonoid QTL were identified with moderate effects (*R* ^2^ < 10%), while a relatively small portion showed major effects (27.4% QTL for BB and 38.2% QTL for ZY with an *R* ^2^ ≥ 10%). The identified QTL in both RIL populations are evenly distributed across the maize genome, and detailed information for the QTL results, including logarithm of odds (LOD) value, 2-LOD confidence interval, explained phenotypic variation (R^2^) of each QTL, as well as candidate genes and their annotations are provided in Additional files 7 and 8: Tables S5 and S6. Two and four flavonoids QTL hot spots were observed across the maize genome in the BB and ZY population, respectively, determined by using 500 permutations at the level of 0.05 (Additional file 5: Figure S2B-S2C; Additional file 7: Table S5). These QTLs were shared by flavonoids that are biochemically related and three known flavonoid pathway genes (*p1*, *c2* and *mrpa3*) located in hot spots on chromosome 1, 4 and 9, respectively (Additional file 5: Figure S2A, S2C).

In Additional file 9: Table S7, the co-localization of QTL and/or significant loci identified across different environments or different populations is summarized. Overall, 49 trait-loci combinations that are 25 QTLs corresponding to 23 traits were detected in more than one environments or populations (AMP, BBRIL, ZYRIL) in this study (Additional file 9: Table S7). Among them, 11 combinations (six loci for 11 traits) were detected in more than two environments which including seven combinations (five loci for seven traits) identified in four environments. Detailed analyses of the candidate genes underlying these loci will almost certainly provide useful further information concerning the flavonoid biosynthetic pathway.

### Candidate genes revealed by multiple evidences

In our previous study, a primary regulatory network consisted of 58 candidate genes for the flavonoid biosynthetic pathway was constructed using an eQTL and qGWAS method based on the expression level of 15 known maize flavonoid pathway genes [47]. Twelve of these 15 genes (namely, *a1*, *bz1*, *bz2*, *c2*, *chi1*, *chi3*, *f3h*, *pr1*, *pac1*, *mrpa3*, *r1*, and *whp1*) were finally involved in the primary network. Moreover, using the same eQTL and qGWAS criteria, a secondary network containing 190 genes was constructed based on the genes present in the primary network. Here, we compared the newly found genes in the primary (46 genes) and secondary network (132 genes) with the candidate genes suggested from GWAS and linkage mapping to identify overlapped genes. Briefly, 11 and 28 genes from the primary and secondary network were found in our genetic mapping results (GWAS or linkage mapping or both), respectively (Table 3).

**Table 3:** Candidate genes of flavonoid biosynthetic pathway revealed by multiple evidences

Environment (Trait)	Candidate Gene	Gene interval (bp)	Annotation
ZYE1(n1555, n1569), ZYE2(n1569)	AC206266.3_FG001*	chr9:17905187-17908271	ATPase activity
ZYE1(n1027), ZYE2(n1581)	AC233883.1_FG006^#^	chr4:193154792-193156631	Unknown
BBE2(n1201), ZYE2(n1240)	GRMZM2G016930*	chr9:16491882-16497920	Hydrolase activity||Serine/threonine-protein phosphatase (EC 3.1.3.16)
BBE1(n1370), AMPE1(n1370), AMPE2(n1569, n1570)	GRMZM2G017536^#^	chr1:47989435-47993634	Unknown
AMPE1(n1263)	GRMZM2G029547*	chr3: 215,757,514-215,764,384	3-beta-hydroxy-delta5-steroid dehydrogenase activity
ZYE2(n1570)	GRMZM2G031938^#^	chr1:245482582-245492124	Antiporter activity
BBE1(n1240)	GRMZM2G032047^#^	chr1:46124872-46126322	Cp protein
BBE1(n1370)	GRMZM2G036086^#^	chr1:47810317-47811419	Peroxisomal biogenesis factor 11 family protein
BBE2(n1173)	GRMZM2G043843^#^	chr3:230536114-230538220	Unknown
BBE2(n1575)	GRMZM2G054013*	chr4:158142358-158147479	Catalytic activity
ZYE2(n1555)	GRMZM2G063550*	chr9:17397398-17399594	Anthocyanidin 3,5-*O*-glucosyltransferase
BBE2(n1575)	GRMZM2G066615^#^	chr4:158338110-158345289	ATP binding
BBE2(n1201), ZYE2(n1240)	GRMZM2G075582^#^	chr9:16654134-16675731	Unknown
BBE1(n1370), AMPE2(n1555, n1562)	GRMZM2G076537^#^	chr1:47509293-47517202	Exonuclease activity binding
ZYE1(n1240), ZYE2(n1027, n1263)	GRMZM2G079471^#^	chr4:195326118-195331420	Unknown
AMPE1(n1581)	GRMZM2G097812*	chr9: 144,508,637-144,511,665	Unknown
BBE2(n1575)	GRMZM2G110192^#^	chr4:159724032-159726475	Unknown
BBE2(n1575)	GRMZM2G110287^#^	chr4:159754943-159757904	Catalytic activity
BBE2(n1270), AMPE1(n1201), AMPE2(n1201)	GRMZM2G114801^#^	chr6:120054604-120057326	Unknown
ZYE1(n1240), ZYE2(n1027, n1263)	GRMZM2G116675^#^	chr4:195200777-195206090	Unknown
BBE2(n1575)	GRMZM2G121075^#^	chr4:158558932-158560342	60S ribosomal protein L12
BBE1(n1370)	GRMZM2G122965^#^	chr1:47699083-47705236	Microtubule motor activity
ZYE1(n1240), ZYE2(n1027, n1263)	GRMZM2G125596^#^	chr4:195381313-195385012	regulation of transcription, DNA-dependent
BBE1(n1240)	GRMZM2G129302^#^	chr1:46863231-46865595	Glycosyltransferase 5
BBE2(n1575)	GRMZM2G147780^#^	chr4:159564598-159569968	Rho guanyl-nucleotide exchange factor activity
ZYE1(n1240), ZYE2(n1027, n1263)	GRMZM2G149843^#^	chr4:195619489-195622261	Vesicle-mediated transport
BBE2(n1201), ZYE2(n1240)	GRMZM2G160463^#^	chr9:16727715-16731614	Unknown
BBE1(n1370), AMPE1(n0511, n1370), AMPE2(n1569, n1570)	GRMZM2G162356^#^	chr1:47900337-47901226	Anaphase-promoting complex subunit 11||zinc ion binding
BBE2(n1270)	GRMZM2G162755*	chr6:119876153-119878032	Anthocyanidin 3-*O*-glucosyltransferase
BBE2(n1270)	GRMZM2G162783*	chr6:119862763-119864524	Transferase activity, transferring hexosyl groups
ZYE2(n1229)	GRMZM2G163671^#^	chr4:195797470-195801122	Maternal Effect Lethal family member||protein binding
ZYE1(n1109, n1370, n1575), ZYE2(n1370, n1581)	GRMZM2G177203^#^	chr10:99379756-99383298	DSBA-like thioredoxin domain containing protein||protein disulfide oxidoreductase activity
BBE1(n1370), AMPE2(n1555, n1562)	GRMZM2G377215^#^	chr1:47504495-47507003	hydrolase activity, hydrolyzing *O*-glycosyl compounds
BBE2(n1575)	GRMZM2G421513^#^	chr4:158560925-158564975	Transcribed locus, weakly similar to NP_001144758.1
BBE1(n1370)	GRMZM2G427418^#^	chr1:47597259-47600865	Transcribed locus, moderately similar to NP_001168327.1
BBE1(n1370)	GRMZM5G807260*	chr1:47827840-47828946	Unknown
BBE1(n1370), AMPE1(n0511, n1109, n1144, n1240, n1562, n1569, n1570), AMPE2(n0511, n1109, n1111, n1144, n1240, n1370)	GRMZM5G819965*	chr1:47906016-47907112	long cell-linked locus protein
ZYE2(n1570)	GRMZM5G842695*	chr1:245310086-245313090	Antiporter activity
ZYE1(n1240), ZYE2(n1027, n1263)	GRMZM5G873295^#^	chr4:195545909-195548933	Unknown
ZYE1(n1111), GWAS(n1111)	GRMZM2G043295	chr9:130524722-130526606	Flavanol-3-*O*-glucosyltransferase
BBE1(n1201), GWAS(n1211)	GRMZM2G059590	chr8:153858072-153864534	Unknown
BBE1(n1216), BBE2(n1216), GWAS(n1216)	GRMZM2G097841	chr10:143746242-143748475	Anthocyanidin reductase
BBE1(n1216), BBE2(n1216), GWAS(n1216)	GRMZM2G097854	chr10:143738314-143740641	Leucoanthocyanidin reductase
BBE1(n1216), BBE2(n1216), GWAS(n1216)	GRMZM2G431504	chr10:143752078-143754011	Leucoanthocyanidin reductase
BBE1(n1518), BBE2(n1518), GWAS(n1518)	GRMZM2G104710	chr10:1919386-1920869	*O*-Methyltransferase
BBE1(n1585), BBE2(n1585), GWAS(n1585)	GRMZM2G104710	chr10:1919386-1920869	*O*-Methyltransferase

* and ^#^represent the genes from primary regulatory network and secondary regulatory network associated with the flavonoid biosynthetic pathway, respectively

In Table 3, we summarized genes for which multiple lines of evidence were provided, i.e., these are genes repeatedly identified in multiple populations or across multiple environments or overlapped genes between the result of network analysis and genetic mapping (Table 3). Three of the 11 genes from the primary network and 14 of the 28 genes from the secondary network mentioned above were detected in more than two environments or for more than two flavonoids in one environment, respectively (Table 3). These genes were subsequently prioritized for further functional characterization. 40% of these 45 candidate genes revealed by multiple evidences mentioned above were annotated as enzymes, while functions of 29% of these genes remain unknown. Genes that were annotated as transcription factor and participating in cellular organization only accounted for a small proportion (Additional file 10: Figure S3).

### Functional verification of candidate genes underlying the natural variation of flavonoids in the mature maize kernel

**Fig. 2:**
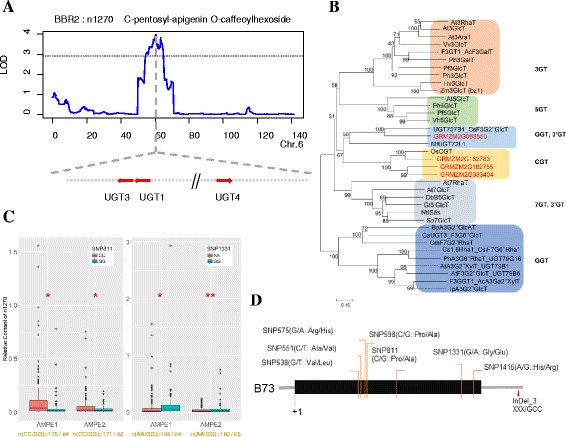
A QTL containing three *UGT*s and re-sequencing and candidate association analysis of *UGT1*. **a** QTL mapping result for the level of *C*-pentosyl-apigenin *O*-caffeoylhexoside (n1270) in the mature maize kernel. LOD values are shown as a function of their genetic positions. And the candidate genes are shown as *red arrows*. **b** Phylogenetic tree of selected flavonoid glycosyltransferases. Candidate genes in the QTL region on chromosome 6 are in *red*. The Genbank accession numbers for the sequences are shown in the parentheses: At3RhaT (NM_102790, At1g30530); At3GlcT (NM_121711, At5g17050); At3AraT (NM_121709, At5g17030); Vv3GlcT (AF000371); AcF3GalT (GU079683); Ph3GalT (AF316552); Pf3GlcT (AB002818); Ph3GlcT (AB027454); Hv3GlcT (X15694); Zm3GlcT (X13501); At5GlcT (NM_117485, At4g14090); Ph5GlcT (AB027455); Pf5GlcT (AB013596); Vh5GlcT (AB013598); CsF3G2″GlcT (HE793682); MtUGT72L1 (EU434684); OsCGT (FM179712); At7RhaT (NM_100480, At1g06000); At7GlcT (NM_129234, At2g36790); DbB5GlcT (Y18871); Gt3′GlcT (AB076697); NtIS5s (AF346431); Sb7GlcT (AB031274); BpA3G2″GlcAT (AB190262); CaUGT3, F3G6″GlcT (AB443870); CmF7G2″RhaT (AY048882); Cs1,6RhaT, CsiF7G6″RhaT (DQ119035); PhA3G6″RhaT, UGT79G16 (Z25802); AtA3G2″XylT, UGT79B1 (NM_124785, At5g54060); AtF3G2″GlcT, UGT79B6 (NM_124780, At5g54010); F3GGT1, AcA3Ga2″XylT (FG404013); IpA3G2″GlcT (AB192315). **c** Boxplot showing the distribution of relative metabolite level (n1270) of lines from the association population with two parental alleles at SNP811 and SNP1331. **d** Sequence polymorphisms between B73 and By804 in *UGT1* (GRMZM2G162755). The SNP identity is indicated by the position starting from the codon ATG. The B73 allele (amino acid) is before the slash, the latter is By804. “XXX” indicates the deletion of three bases. Abbreviations: A3G, anthocyanin 3-*O*-glucoside; A3Ga, anthocyanin 3-*O*-galactoide; F3G, flavonol 3-*O*-glucoside; F7G, flavonoid 7-*O*-glucoside; 3AraT, 3-*O*-arabinosyltransferase; 3GlcT, 3-*O*-glucosyltransferase; 3′GlcT, 3′-*O*-glucosyltransferase; 3GalT, 3-*O*-galactosyltransferase; 3RhaT,3-*O*-rhamnosyltransferase; 5GlcT, 5-*O*-glucosyltransferase; 7GlcT, 7-*O*-glucosyltransferase; 7RhaT, 7-*O*-rhamnosyltransferase; 2″GlcT, 2″-*O*-glucosyltransferase; 2″RhaT, 2″-*O*-rhamnosyltransferase; 2″XylT, 2″-*O*-xylosyltransferase; 6″RhaT, 6″-*O*-rhamnosyltransferase; CGT, *C*-glucosyltransferase; NtIS5a, salicylate-induced glucosyltransferase. Abbreviations for species: Ac, Actinidia chinensis; At, Arabidopsis thaliana; Bp, Bellis perennis; Cm, Citrus maxima; Csa, Crocus sativus; Csi, Citrus sinensis; Db, Dorotheanthus bellidiformis; Gt, Gentiana triflora; Hv, Hordeum vulgare; Ip, Ipomoea purpurea; Nt, Nicotiana tabacum; Os, Oryza sativa; Pf, Perilla frutescens; Ph, Petunia hybrida; Sb, Scutellaria baicalensis; Vh, Verbena hybrida; Vv, Vitis vinifera; Zm, Zea mays [80]

According to the mapping results and multiple information regarding prior knowledge of flavonoid biosynthesis and functional annotation of candidate genes, we chose several genes that were supported by multiple evidences for further verification. A QTL on chromosome 6 was identified for the level of *C*-pentosyl-apigenin *O*-caffeoylhexoside (n1270) in the B73/BY804 RIL population (Fig. 2a). Three genes, GRMZM2G162755 (*UGT1*, chr6:119876153-119878032), GRMZM2G162783 (*UGT3*, Chr6:119,862,763-119,864,524) and GRMZM2G383404 (*UGT4*, chr6:120018887-120020772) which are all annotated as flavonoids 3-*O*-glucosyltransferase are located within this QTL. *UGT1* is about 12Kb upstream of *UGT3*, and both genes were identified as targets of the R2R3-MYB transcript factor *P1* [31]. *UGT1* co-expressed with several genes involved in the flavonoid pathway, such as *C2* (GRMZM2G422750, chalcone synthase), *Chi1* (GRMZM2G155329, chalcone flavanone isomerase 1) and *Pr1* (GRMZM2G025832, cytochrome P450) [47]. Moreover, *UGT1*, *UGT3* and *UGT4* all show sequence similarity with the rice *C*-glycosyltransferase (*OsCGT*, Fig. 2b). However, only *UGT3* has been putatively identified, on the basis of homology, to be a bifunctional *C*- and *O*-glycosyltransferase, and *UGT1* and *UGT2* (another *UGT* on chromosome 9) is unable to produce apigenin 6-*C*-glucoside following supply of 2-hydroxynaringenin as a flavonoid receptor [29]. The function of *UGT4* in the flavonoid biosynthesis has, however, been investigated using a re-sequencing and candidate gene association method, whereby some proposed functional variations were revealed [44].

Herein we looked into the genetic variations between the two parental lines of the BB RIL population (B73 and By804) and found seven SNPs between the parents in the coding sequence of *UGT1*, which could cause nonsynonymous mutations (Fig. 2d). Three pairs of KASP (LGC) primers which can successfully genotype three (i.e., SNP811, SNP1331, and SNP1415) of the seven SNPs were used to test the association panel aiming to validate the function of these three SNPs in *UGT1*. They all exhibited a minor allele frequency (MAF) of more than 0.05. At the sites SNP811 (a Pro to Ala variant) and SNP1331 (a Gly to Glu variant), phenotypic values of lines with the alleles from the two parents were significantly different (t test, *P* < 0.05; Fig. 2c). Significant phenotypic differences between the lines harboring B73 alleles and By804 alleles were also observed for several other flavonoids detected in this study. For instance, the levels of three apigenin derivatives, chrysoeriol and six chrysoeriol derivatives, four tricin derivatives and cyanidin 3-*O*-glucoside detected in lines with two parental alleles at SNP811 were significantly different. Similarly, the levels of cyanidin 3-*O*-glucoside, chrysoeriol di-*C*-hexoside, 3′,4′,5′-tricetin-*O*-hexoside and apigenin *C*-pentosyl-*O*-coumaroylhexoside in lines with two parental alleles at SNP1331 were significantly different (Additional file 11: Figure S4). In addition, we conducted candidate association analysis using these three SNPs - SNP1331 displayed the lowest *P* value and can therefore be considered as the most promising functional site among these three SNPs (Additional file 12: Table S8). Compared to SNP811, SNP1331 was associated with more flavonoids, which may suggest that it exhibits broader substrate specificity.

**Fig. 3:**
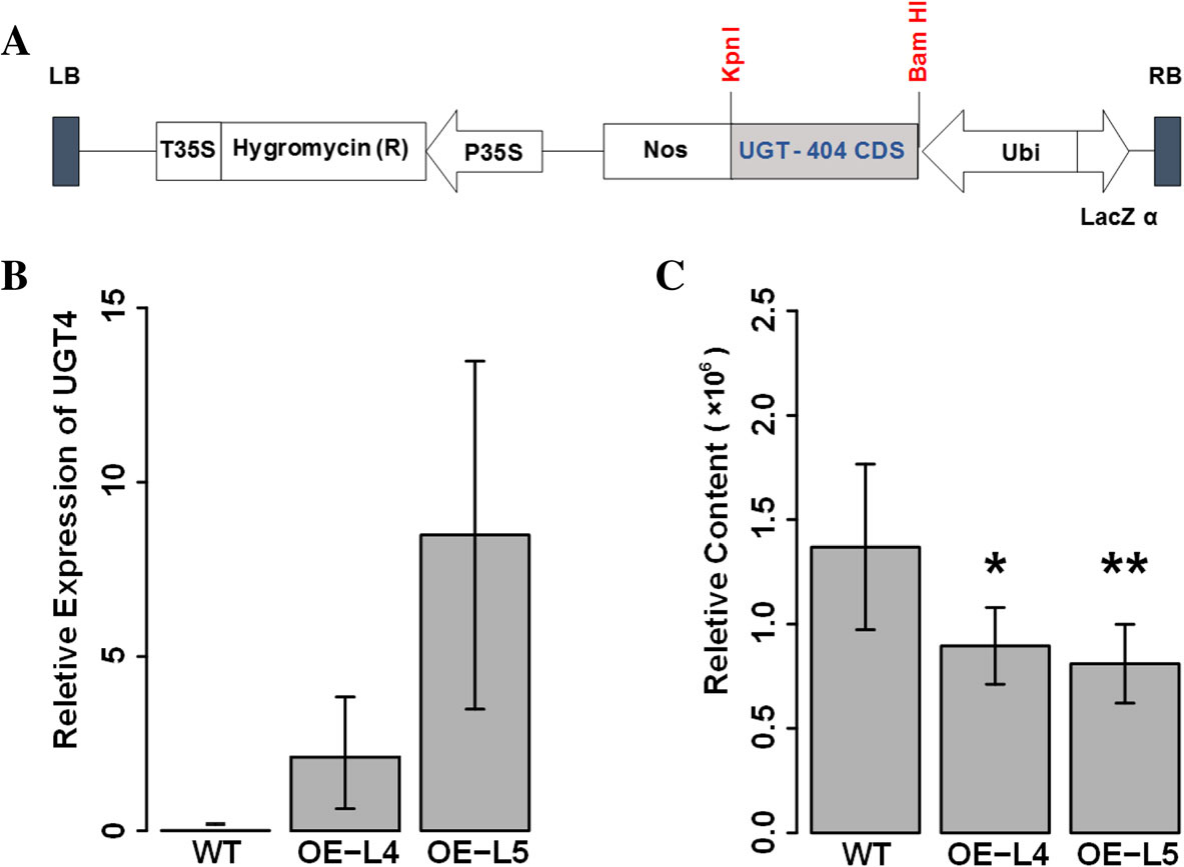
Transgenic result of *UGT4*. **a** Diagram of over-expression construct. **b** The bar plot showing the average mRNA level of *UGT4* (GRMZM2G383404) in the wild type (WT) and over-expression lines (T1) (the individual number is 9, 5, 9 for WT, L4 and L5, respectively, 3 technical replicates for each line). **c **The bar plot showing the relative contents of apigenin *C*-pentosyl-*C*-pentoside (n1201) in the WT and *UGT4* over-expression lines (*n* = 9, 5, 9 for WT, L4 and L5, respectively), * and ** represent the significant level of *P* < 0.05 and *P* 0.01, respectively

GRMZM2G383404 (*UGT4*) is around 142 kb away from *UGT1*, which is associated with the level of apigenin *C*-pentosyl-*C*-pentoside (n1201) as revealed by our previous genome wide association analysis, and an amino acid substitution (Asp to Ala) was suggested as one of the functional genetic variants [44]. In the present study, we generated over-expression lines by ectopically expressing GRMZM2G383404 under the control of the maize ubiquitin promoter in the rice cultivar Zhonghua11 (Fig. 3a). We detected the level of flavonoids in the rice leaves of the wild type and T1 individuals of two over-expression lines (L4 and L5) (Fig. 3b). The level of more than half (14/26) of the detected flavonoids were significantly decreased in the over-expression lines. The fold change of these 14 flavonoids between the over-expression lines and wild type ranged from 0.25 to 0.68 (Fig. 3c and Additional file 13: Figure S5). Among them, fold change between the over-expression lines and wild type of the level of apigenin *C*-pentosyl-*C*-pentoside was around 0.65. Along with apigenin *C*-pentosyl-*C*-pentoside, two other apigenin derivatives (i.e., apigenin 7-*O*-glucoside and apigenin di-*C*-hexoside) were also affected (Additional file 13: Figure S5). Notably, the level of all the tricin derivatives detected here (and tricin itself) was significantly decreased (Additional file 13: Figure S5). Moreover, the content of chrysoeriol, chrysoeriol *O*-hexoside and vitexin were also significantly decreased (Additional file 13: Figure S5). However, no significant changes were found for the content of *C*-pentosyl-apigenin *O*-caffeoylhexoside, for which *UGT4* was identified in the QTL region as mentioned above. Hence, *UGT1* and *UGT3* but not *UGT4* could be the causative genes for the variance of *C*-pentosyl-apigenin *O*-caffeoylhexoside. However, the transgenic result of *UGT4* can suggest its influence in the flavonoid biosynthesis. However, further biochemical assay is needed to strongly confirm the function and activity.

**Fig. 4:**
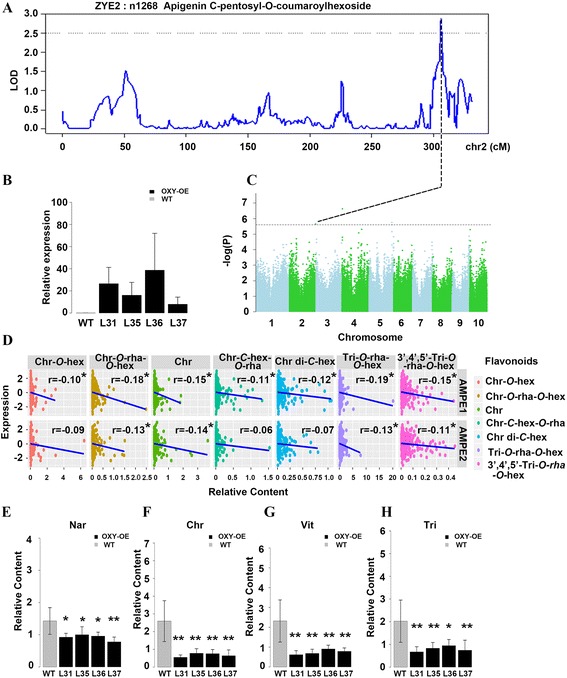
Linkage and association mapping of *OXY* and validation by transformation. **a** and **c** Diagram of linkage mapping and GWAS results for the level of Apigenin *C*-pentosyl-*O*-coumaroylhexoside in maize kernel. LOD values are shown as a function of their genetic positions. And the peak SNP is located within *OXY* (GRMZM5G843555). **b** The bar plot showing the mRNA level of *OXY* in wild type (WT) and over-expression lines (T1) (the individual number is 6, 10, 7, 8 for WT, L31, L35, L36 and L37, respectively, 3 technical replicates for each line individual). **d** Plot of correlation between the content of different flavonoids and the normalized expression level of gene *OXY* in association panel*.* **e**-**h** The bar plot for the relative contents (fold change relative to the mean level of each flavonoid) of naringenin, chrysoeriol, vitexin and tricin between the WT and *OXY* over-expression lines (*n* = 6, 10, 10, 10 respectively). * and ** represent the significant level of *P* < 0.05 and *P* < 0.01, respectively

On chromosome 2, gene GRMZM5G843555 was suggested to be important in determining the level of apigenin *C*-pentosyl-*O*-coumaroyl hexoside (n1268) by both linkage mapping in Zong3/Yu87-1 population (Fig. 4a) and GWAS in AMPE1 (Fig. 4c). GRMZM5G843555 is annotated as an oxoglutarate/iron-dependent oxygenase (*OXY*), which belongs to the oxygenase superfamily. However, GRMZM5G843555 (*OXY*) shows low sequence similarity with the well-known 2-ODD genes, such as FNS. *OXY* is one of the maize prolyl 4-hydroxylase family (P4Hs) members, which may play a role in tolerance to abiotic stresses, such as water-logging [48]. Correlations between the content of various flavonoids and the expression level of *OXY* revealed that the content of chrysoeriol, chrysoeriol *O*-rhamnosyl-*O*-hexoside, tricin *O*-rhamnosyl-*O*-hexoside and 3′,4′,5′-tricetin *O*-rhamnosyl-*O*-hexoside, chrysoeriol *O*-hexoside, chrysoeriol di-*C*-hexoside and chrysoeriol *C*-hexosyl-*O*-rhamnoside were negatively correlated with expression level of *OXY* (*r* = -0.19 ~ -0.1; *p* < 0.05) (Fig. 4d). We further profiled the rice over-expression lines and quantified the level of 26 flavonoids (Fig. 4b). The levels of 20 flavonoids were significantly decreased compared with that of the wild type (Fig. 4e-h, Additional file 14: Figure S6). Within these 20 flavonoids, the content of six flavonoids was negatively correlated with the *OXY* expression level. Based on the result, we speculate that the *OXY* may act as a competitor or inhibitor of the flux through the apigenin, chrysoeriol and tricin branches of flavonoid metabolism.

In addition, abundant genetic variants between Zong3 and Yu87-1 in the promoter region of *OXY* were observed (Additional file 15: Figure S7). *Cis*-element prediction using PlantCARE (http://bioinformatics.psb.ugent.be/webtools/plantcare/html/) found a variant between the sequences of the two parental lines at the MBS II (MYB binding site II, [49]). The variant at this binding site may affect the function of *OXY* through transcriptional regulation, as suggested by the finding in *Petunia Hybrida* [49]. Indeed, a strong *cis*-eQTL for *OXY* was identified in our previous study, which may suggest the potential function of this genetic variant at the upstream of this gene (Additional file 16: Figure S8).

**Fig. 5:**
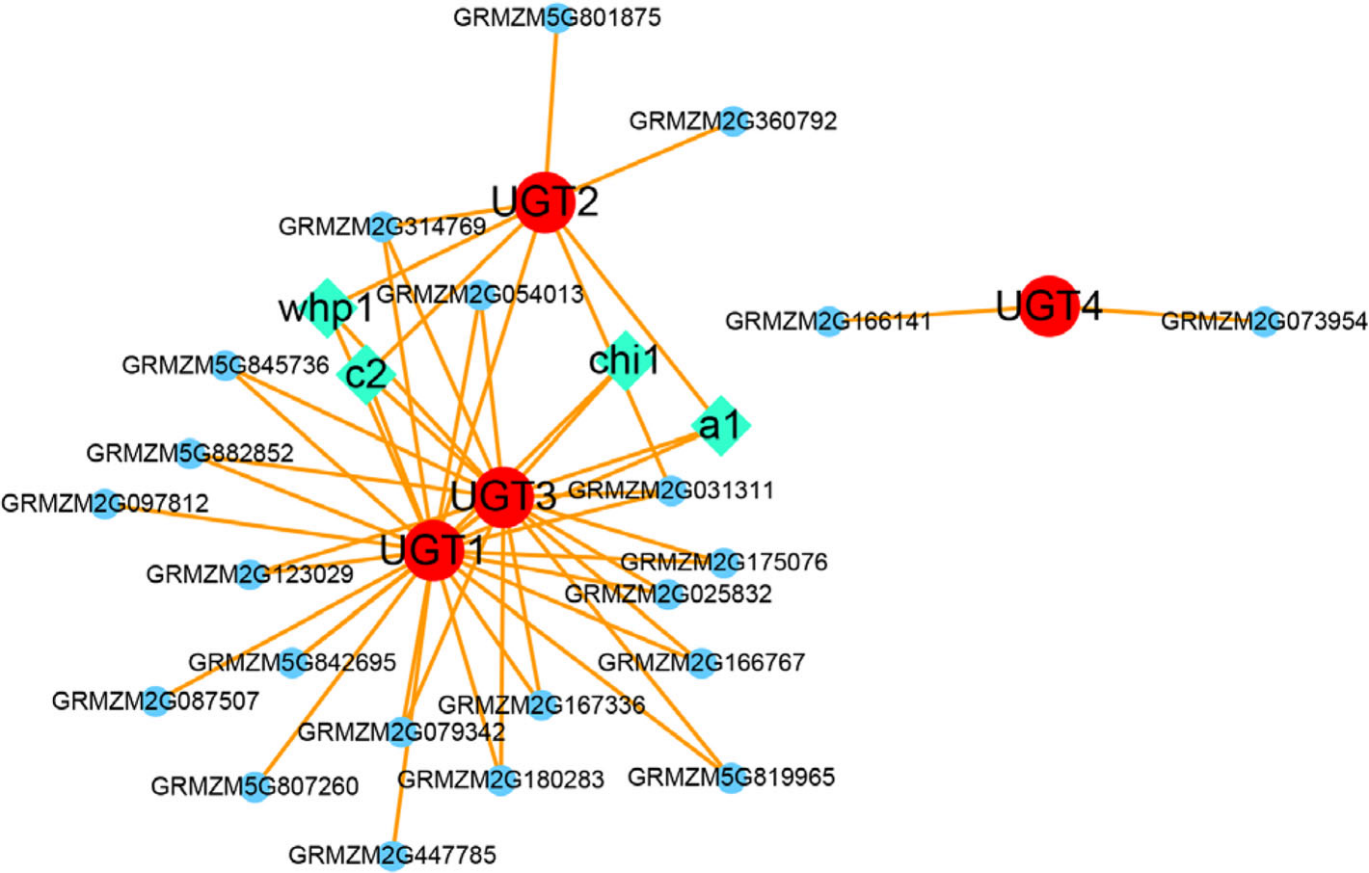
Co-expression network for *UGT1*, *UGT2*, *UGT3* and *UGT4* based on a qGWAS method. The *red* indicates these four genes. The *green diamond* indicates the known enzyme involved in the flavonoid pathway. The *blue circle* indicates the uncharacterized co-expressing genes

To investigate the co-expression mode of the above mentioned genes, a qGWAS-based network was constructed (Fig. 5). GRMZM5G843555 (*OXY*) is not in this network for no related genes found by using the threshold of *P* < 3.5 × 10^−7^ (0.01/28369). *UGT4* clustered independently from the rest. Four well-known genes involving in the flavonoids biosynthesis are present in the co-expression network, such as *a1* (GRMZM2G026930), *c2* (GRMZM2G422750), *chi1* (GRMZM2G155329) and *whp1* (GRMZM2G151227). 22 uncharacterized genes are also revealed in the network, including a gene homologous to chalcone isomerase (GRMZM2G175076) and other 21 genes with unknown function or without direct functional annotations related to flavonoid biosynthesis.

## Discussion

Metabolomics, which promotes the study of plant metabolism, offers the capacity to speed up the breeding process toward high yielding and nutritional crops [50, 51]. With the advent of high-efficiency metabolic profiling and high-throughput sequencing technologies, studies of genetic dissection of metabolomics diversity based on GWAS and linkage analysis have been reported recently in several plant organisms such as maize [44, 47, 52], rice [41, 53], tomato [54–57] and *Arabidopsis thaliana* [58, 59]. In addition to the dataset generated from our previous untargeted metabolomics-based genetic mapping, here we focus on the flavonoids that were found in the mature kernel harvested from an association panel and two RIL populations grown across multiple environments [44, 47]. Based on a combined analysis (genetic mapping, metabolite profiling and gene regulatory network analysis), we firstly dissected the genetic control of the variation of 29 flavonoids. This indicated that few loci with large effect (with *R* ^*2*^ > 20%) along with more loci with minor to modest genetic contribution underlie the variation of flavonoids level in the mature maize kernel, and the average effect that each locus contributes is modest (*R* ^*2*^ is ~10%). Since maize kernel is a storage tissue, we would assume that more loci responsible for flavonoid variation can be identified in the mature kernel as compared with vegetative tissues such as leaves. It will however be worthwhile to investigate the accumulation and distribution of flavonoids in diverse tissue types in further research. Secondly, we are able to propose dozens of promising candidate genes involved in maize flavonoid biosynthesis. Specifically, three *UGT*s which are all evolutionary close to rice *CGT* (Fig. 2b), are located in one glycosylated flavone QTL region (Fig. 2a). The function of *UGT3* was elucidated as having both *C*- and *O*- glucosyltransferase ability [29], while the biochemical activity of *UGT1* and *UGT4* remains unknown. Re-sequencing the two parental lines as well as the whole association panel and subsequent candidate association analysis have provided us with the potential functional genetic variants of *UGT1*. However, whether and if so how the two amino acid replacements in the coding region of *UGT1* influence the protein structure and enzymatic activity remains to be answered. In addition given the genomic location of these three *UGT* genes, it will also be interesting to look into the genomic divergence of the region covering the *UGT1* locus between diverse maize germplasm (including inbred lines, landraces and wild progenitors) from an evolutionary perspective. On the other hand, we found significant decreases of several flavonoids in *UGT4* overexpressing rice leaves. And in our co-expression network, *UGT1*, *UGT2* and *UGT3* are clustered with four well-characterized flavonoids biosynthesis pathway genes, while *UGT4* clustered independently from the rest. Further molecular and biochemical evidence will be needed to provide the exact mechanism underlying this observation. In addition, the maize *OXY* gene exerted a negative influence on the levels of a broad range of flavones in the over-expressing rice leaves.

It has been documented that flavonoids have potent bioactivities which are beneficial for human health. Epidemiologic studies have suggested that a diet rich in flavones exhibits some anti-carcinogenic as well as anti-angiogenic and anti-inflammatory bioactivities [60–62]. Flavonoids also have antioxidant and anti-diabetic potential when added to food [63]. Furthermore, flavonoids also play an important role in protecting the plant itself against biotic and abiotic stresses [64]. In particularly, the *C*- glycosyl flavone has been revealed to participate in protection against UV-B radiation and defense against pathogens [30, 65]. It is thus essential to find functional genes and understand their regulatory networks as an approach for biofortification of these valuable compounds in maize [12, 47, 64]. The genes *OXY* and *UGTs* investigated in this study are promising targets for further crop flavonoid content improvement through favorable allele pyramiding or metabolic engineering techniques on the basis of intensive allele mining and a full understanding of gene regulatory network. The non-targeted metabolomics approach, as adopted here, has the potential to enable the findings of novel genes and pathways. To realize this in the future, an elaborate design which takes into account biological or physiological context would also be helpful.

## Conclusions

In our current study, we explored the genetic influences on the flavonoid biosythensis in maize kernel based on integrating the genomic, transcriptomic and metabolomic information which provided a rich source of potential candidate genes. As indicated here and in our previous study [44], the integrated genomics based genetic mapping strategy is highly efficient for defining the complexity of functional genetic variants and their respective regulatory networks as well as in helping to select candidate genes and allelic variance before embarking on laborious transgenic validations. In a similar vein, a maize protoplast complementation system developed by Casas et al (2016) was very recently proposed [12] as a means to probe the activity of metabolic enzymes in an approach that circumvents the need for transgenic plants. This method as well as the approach we took in the current study offers new opportunities to advance beyond the QTL/association mapping approach and towards a complete understanding of maize flavonoid biosynthesis.

## Methods

### Plant materials, genotypic and metabolic data

The metabolic data used in this study is measured from genetic materials including an association mapping panel (referred to as AMP hereafter) for GWAS and two Recombinant Inbred Lines populations (RILs; BB and ZY) for linkage analysis as described previously [44, 47, 66, 67]. The AMP consisted of 368 diverse inbred lines and were planted in Yunnan (Kunming, E 102°30′, N 24°25′, referred to as AMPE1 hereafter) and Chongqing (E 106°50′, N 29°25′, referred to as AMPE2 hereafter) in March of 2011, respectively. The 197 BB RIL population derived from a cross between B73 and a high-oil line By804 were planted in Hainan (Sanya; E 109°519, N 18°259) in October of 2010 (referred to as BBE1 hereafter) and Henan (Zhengzhou; E 113°429, N 34°44′) in June of 2011 (referred to as BBE2 hereafter). The 197 lines that were derived from the cross between Zong3 and Yu87-1 were planted in Yunnan (Kunming; E 102°309, N 24°259; referred to as ZYE1 hereafter) and Henan (Zhengzhou; E 113°429, N 34°449; referred to as ZYE2 hereafter) in March and June of 2011, respectively. All the inbred lines were planted in one-row plots in an incompletely randomized block design. All lines were self-pollinated and ears of each plot were hand-harvested, followed by air drying and shelling. For each line, ears from five plants were harvested at the same maturity and bulked. Twelve well grown kernels were randomly selected from the harvested ears and bulked for grinding. Samples were extracted before analysis using an LC-ESI-MS/MS system [44, 47].

All 368 diverse maize inbred lines of the AMP had been genotyped by the Illumina MaizeSNP50 BeadChip with 56,110 genome-wide SNPs and RNA sequencing on the immature kernels of 15 days after pollination, which resulted in the genotypic data of 556,809 high-quality SNPs with MAF > 0.05 across the maize genome [68, 69]. Both RIL populations were genotyped using the Illumina MaizeSNP50 BeadChip containing 56,110 SNPs [70] and linkage map was constructed using recombinant bins for both RIL populations. Briefly, a map containing 2,496 and 3,071 unique bins was constructed for BB and ZY RILs, respectively [71].

### Genetic mapping

Genome-wide association study (GWAS) was performed using a compressed mixed linear model (cMLM) implemented in the software TASSEL 3.0, accounting for the population structure (Q) and familial relationship (K) [72]. SNPs with minor allele frequency (MAF ≥ 5%) in the 368 lines were employed in the association analysis. To facilitate the interpretation of GWAS results, P value of each SNP was calculated and significance was defined at a uniform threshold of 1.8 × 10^−6^ (i.e., P ≤ 1/N, *N* = 556809 which is roughly a Bonferroni correction). SNP with the lowest P value (i.e., the lead SNP) and its corresponding gene were reported for each significant flavonoid locus (see Additional file 4: Table S3). Linkage mapping was conducted using composite interval mapping method [73] implemented in Windows QTL Cartographer V2.5 for each flavonoid trait identified in both RIL populations [74]. Zmap (model 6) with a 10-cM window and a walking speed of 0.5 cM was used. To determine a threshold for significant QTLs, 500 permutations (*P* = 0.05) were used for each flavonoid identified in both RIL populations. The bins were clearly defined, and a uniform LOD value was assigned for each bin. The confidence interval for each QTL was assigned as a 2 LOD drop from the peak. The setting of parameters was the same as described previously [47]. Detailed information including physical location, confidence interval, and *R* ^2^ (explained phenotypic variance) of each QTL for each flavonoid trait is shown in Additional file 7: Table S5. To test the cross-validation between GWAS and linkage analysis, 200 kb region of loci identified by GWAS (the 100 kb upstream and downstream region of the lead SNP) was compared with the physical region of QTL.

### Candidate gene identification

The filtered working gene list of maize genome was downloaded from MaizeGDB (http://www.maizegdb.org) to identify possible candidate genes in each QTL. Candidate genes were annotated according to InterProScan (http://www.ebi.ac.uk/interpro/scan.html). All potential candidate genes and their annotations within 100 kb (50 kb upstream and downstream of the lead SNP) of the loci identified from GWAS are listed in Additional file 6: Table S4. Candidate genes associated with the corresponding flavonoid trait that were searched within the confidence interval for each QTL from linkage mapping are listed in Additional file 8: Table S6. The most likely candidate gene was selected by testing for either gene flavonoid association or association between the gene and pathway. For the loci without appropriate candidates, the gene nearest to the lead SNP is assigned.

### Constructs and transformation

To generate *OXY* over-expressing constructs, the vector pCAMBIA1301s which contains the selectable marker gene *hpt* and 35S promoter was used. There are six transcripts for *OXY* on the B73 reference genome. To decide which one to be transferred, five genotypes with highest n1268 content and five with lowest n1268 content were chosen to compare the abundance between different transcripts. We observed that transcript T04 with six exons was the most highly expressed. So the B73 genomic DNA fragment of *OXY* T04 (from the ATG to TGA) was amplified and the PCR product was cloned into vector pCAMBIA1301s with restriction enzyme KpnI and XbaI. On the other hand, gene *UGT4* has only one transcript on B73 genome. Therefore *UGT4* T01 DNA fragment of B73 (from ATG to TGA) was amplified to generate the *UGT4* over-expressing construct into vector pCAMBIA 1300nu (with the selectable marker gene *hpt*) using restriction enzyme BamHI and KpnI. The final plant expression vector was introduced into Agrobacterium EHA105 by electroporation and calli induced from mature seeds of an elite *japonic*a rice cultivar Zhonghua11 were used for Agrobacterium-mediated transformation [75].

### Expression analyses

We isolated total RNA from rice leaves with TRIzol (Invitrogen) as the manufacturer’s instructions. The first-strand cDNA was synthesis using a TransScript One-Step gDNA Removal and cDNA Synthesis SuperMix (TansGen Bioteke) according to the manufactures’ protocol. Quantitative PCR was performed on an optical 96-well plate in a BioRAD PCR system (CFX96) with SYBR Mix (Vazyme). The relative expression level of gene *OXY* and *UGT4* was determined with the rice Actin as an internal control. The expression measurements were obtained using the relative quantification method [76]. The leaves of transgenic rice are sampled and stored immediately in liquid nitrogen. The following extraction and flavonoid profiling were as described previously [44]. A student’s t-test was applied to examine the difference between over-expression line and the wild line.

### Phylogenetic analysis

UGT protein sequences were aligned using CLUSTAL W implemented in MEGA7 (version 7, http://www.megasoftware.net/) [77]. A phylogenetic tree was constructed from aligned UGT protein sequences by MEGA7 using the neighbor-joining method [78] with the following parameters: bootstrap method (1000 replicates), Poisson model, uniform rates, and complete deletion.

### Re-sequencing and allele identification

For *UGT1* (GRMZM2G162755) and *OXY* (GRMZM5G843555), the PCR fragments from gDNA of B73 and By804 were sequenced via the Sanger re-sequence method. The SNPs were identified using CLUSTAL OMEGA online (http://www.ebi.ac.uk/Tools/msa/clustalo/). We designed one of the two direction primers with 3′end at the SNPs of *UGT1* according to the direction provided by LGC (Laboratory of the Government Chemist) and performed PCR with the KASP Assay Mix referring to its procedure. All primers for vector construction and re-sequencing used in this study are shown in Additional file 17: Table S9.

### Construction of co-expression network

A qGWAS-based method was adopted to construct the co-expression network as described previously [47]. The expression data was obtained from our previous RNA sequencing analysis on maize kernels (at the stage of 15 day after pollination) of the association panel containing 368 maize inbred lines [68]. We focused on the four candidate genes (*UGT1*, *UGT2*, *UGT3*, and *UGT4*) and their co-expressing genes with the threshold of *P* < 3.5 × 10^−7^(0.01/28369). The program Cytoscape [79] was used to display the network.

## Additional files

Additional file 1: Table S1. Flavonoid intensities of each line in AMP and both BB and ZY populations planted across multiple environments. (XLS 490 kb)
Additional file 2: Table S2. Range and mean of fold changes of flavonoid traits measured in AMP and both BB and ZY populations. (XLS 23 kb)
Additional file 3: Figure S1. Distribution of the heritability of Flavonoids. (PDF 18 kb)
Additional file 4: Table S3. List of significant loci identified by GWAS and their detailed information. (XLS 218 kb)
Additional file 5: Figure S2. Chromosomal distribution of Flavonoid QTLs identified in this study. (PDF 899 kb)
Additional file 6: Table S4 List and detailed information of candidate genes within the significant loci identified by GWAS. (XLS 101 kb)
Additional file 7: Table S5. Detailed information of QTLs identified for each flavonoid in both BB and ZY populations across two environments. (XLS 63 kb)
Additional file 8: Table S6. List of candidate genes and their detailed information in the peak bin for each QTL. (XLS 1243 kb)
Additional file 9: Table S7. Overview of the co-localization of QTLs identified across different environments or different populations. (XLS 29 kb)
Additional file 10: Figure S3. Classification of candidate genes according to their functional annotations. (PDF 32 kb)
Additional file 11: Figure S4. Boxplots showing the distribution of flavonoids level. (PDF 158 kb)
Additional file 12: Table S8. Result of candidate gene association analysis on GRMZM2G162755. (XLS 29 kb)
Additional file 13: Figure S5 Bar plot of the relative flavonoid levels (fold change relative to the mean level of each flavonoid) that are significantly different between the wild type (WT) and *UGT4* over-expression lines. (PDF 141 kb)
Additional file 14: Figure S6. Bar plot of the relative flavonoid levels (fold change relative to the mean level of each flavonoid) that are significantly different between the wild type (WT) and *OXY* over-expression lines. (PDF 131 kb)
Additional file 15: Figure S7. The sequence polymorphisms between B73 and By804 of the promoter region of gene *OXY*. (PDF 215 kb)
Additional file 16: Figure S8. Manhattan plot showing the GWAS result of the expression level of *OXY* (GRMZM5G843555). (PDF 59 kb)
Additional file 17: Table S9. All primers used in the study. (XLS 26 kb)

## Abbreviations

2″GlcT: 2″-*O*-glucosyltransferase
2″RhaT: 2″-*O*-rhamnosyltransferase
2″XylT: 2″-*O*-xylosyltransferase
2-ODDs: Oxoglutarate-dependent dioxygenases
3′GlcT: 3′-*O*-glucosyltransferase
3AraT: 3-*O*-arabinosyltransferase
3GalT: 3-*O*-galactosyltransferase
3GlcT: 3-*O*-glucosyltransferase
3RhaT: 3-*O*-rhamnosyltransferase
5GlcT: 5-*O*-glucosyltransferase
6″RhaT: 6″-*O*-rhamnosyltransferase
7GlcT: 7-*O*-glucosyltransferase
7RhaT: 7-*O*-rhamnosyltransferase
*a1*: *Anthocyaninless 1*
A3G: Anthocyanin 3-*O*-glucoside
A3Ga: Anthocyanin 3-*O*-galactoide
Ac: Actinidia chinensis
Ala: Alanine
AMP: Association mapping panel
At: Arabidopsis thaliana
ATP: Adenosine triphosphate
BGLU6: Beta glucosidase 6
bHLH: Basic Helix-Loop-Helix
Bp: Bellis perennis
*bz1*: *Bronz 1*
*bz2*: *Bronz 2*
*C2*: *Colorless 2*
CGT: *C*-glucosyltransferase
CHI: Chalcone isomerase
*chi1*: *Chalcone isomerase 1*
*chi3*: *Chalcone isomerase 3*
CHS: Chalcone synthase
Cm: Citrus maxima
cMLM: Compressed mixed linear model
Csa: Crocus sativus
Csi: Citrus sinensis
Db: Dorotheanthus bellidiformis
eQTL: Expression quantitative trait loci
F2H: Flavanone 2-hydroxlase
F3G: Flavonol 3-*O*-glucoside
F3GG7R: Flavonol 3-*O*-gentiobioside 7-*O*-rhamnoside
*f3h*: *Flavanone 3-hydroxylase*
F7G: Flavonoid 7-*O*-glucoside
FLS1: Flavonol synthase
FNS I: Flavone synthase I
FNSII: Flavone synthase II
GCA: Chlorogenic acid
Glu: Glutamic acid
Gly: Glycine
Gt: Gentiana triflora
GWAS: Genome wide association
H^2^: Broad-sense heritability
Hv: Hordeum vulgare
Ip: Ipomoea purpurea
LC-MS/MS: Liquid chromatography tandem mass spectrometry
LOD: Logarithm of odds
MAF: Minor allele frequency
MBS II: MYB binding site II
NADPH: Nicotinamide adenine dinucleotide phosphate
NILs: Near isogenic lines
Nt: Nicotiana tabacum
NtIS5a: Salicylate-induced glucosyltransferase
Os: Oryza sativa
OXY: Oxoglutarate/iron-dependent oxygenase
*P1*: *Pericarp1*
P4H: Prolyl 4-hydroxylase
*pac1*: *Pale aleurone color 1*
Pf: Perilla frutescens
Ph: Petunia hybrida
*pr1*: *Red aleurone 1*
Pro: Proline
qGWAS: Quantitative genome-wide association study
QTL: Quantitative trait locus
r1: *Red 1*
R^2^: The percentage of phenotypic variation
RIL: Recombinant inbred line
Sb: Scutellaria baicalensis
*sm1*: *Salmon silks 1*
*sm2*: *Salmon silks 2*
TF: Transcription factor
UDP: Uridine diphosphate
UGT: UDP-glycosyltransferases
Vh: Verbena hybrida
Vv: Vitis vinifera
*whp1*: *White pollen 1*
Zm: Zea mays
