# Supplementary material for: Integrated genomics-based mapping reveals the genetics underlying maize flavonoid biosynthesis
Source: BMC Plant Biol. 2017 Jan 18;17:17. doi: 10.1186/s12870-017-0972-z (PMC5242060; doi:10.1186/s12870-017-0972-z)
Supplement: Additional file 3: Figure S1. — Distribution of the heritability of Flavonoids. (PDF 18 kb) [file 12870_2017_972_MOESM3_ESM.pdf]

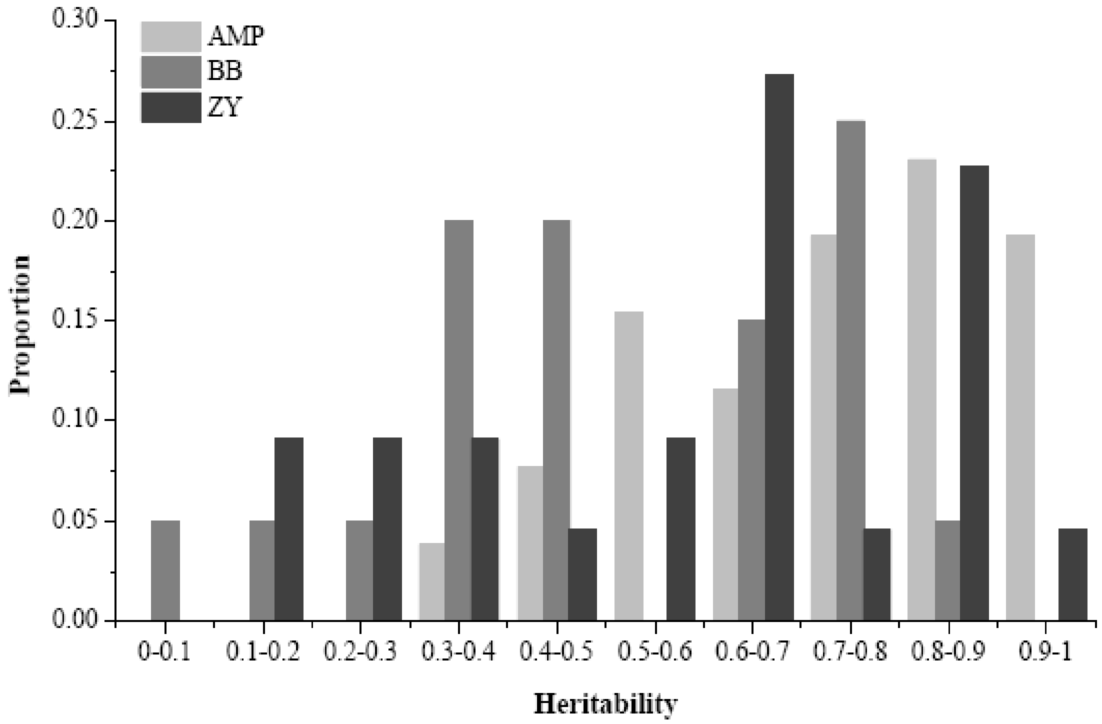

Figure S1. Distribution of the heritability of flavonoids. The vertical bars show the proportion of flavonoids that were detected in both experiments of AMP and each RIL population.
