# Supplementary material for: Integrated genomics-based mapping reveals the genetics underlying maize flavonoid biosynthesis
Source: BMC Plant Biol. 2017 Jan 18;17:17. doi: 10.1186/s12870-017-0972-z (PMC5242060; doi:10.1186/s12870-017-0972-z)
Supplement: Additional file 5: Figure S2. — Chromosomal distribution of Flavonoid QTLs identified in this study. (PDF 899 kb) [file 12870_2017_972_MOESM5_ESM.pdf]

A

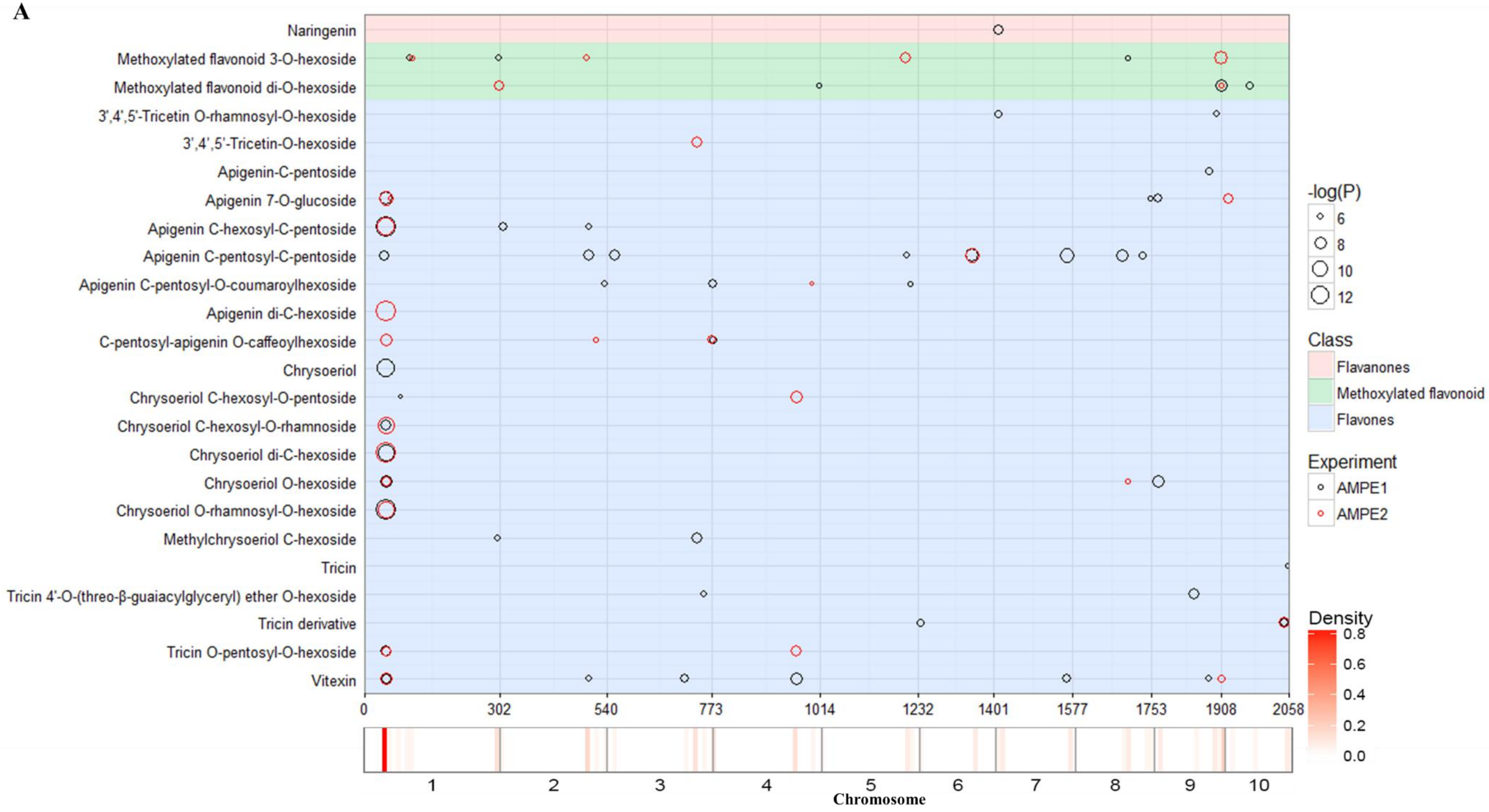

Naringenin
Methoxylated flavonoid 3-O-hexoside
Methoxylated flavonoid di-O-hexoside
3',4',5'-Tricetin O-rhamnosyl-O-hexoside
3',4',5'-Tricetin-O-hexoside
Apigenin-C-pentoside
Apigenin 7-O-glucoside
Apigenin C-hexosyl-C-pentoside
Apigenin C-pentosyl-C-pentoside
Apigenin C-pentosyl-O-coumaroylhexoside
Apigenin di-C-hexoside
C-pentosyl-apigenin O-caffeoylhexoside
Chrysoeriol
Chrysoeriol C-hexosyl-O-pentoside
Chrysoeriol C-hexosyl-O-rhamnoside
Chrysoeriol di-C-hexoside
Chrysoeriol O-hexoside
Chrysoeriol O-rhamnosyl-O-hexoside
Methylchrysoeriol C-hexoside
Tricin
Tricin 4'-O-(threo-β-guaiacylglyceryl) ether O-hexoside
Tricin derivative
Tricin O-pentosyl-O-hexoside
Vitexin
0
302
540
773
1014
1232
1401
1577
1753
1908
2058
1
2
3
4
5
6
7
8
9
10
Chromosome
-log(P)
6
8
10
12
Class
Flavanones
Methoxylated flavonoid
Flavones
Experiment
AMPE1
AMPE2
Density
0.8
0.6
0.4
0.2
0.0

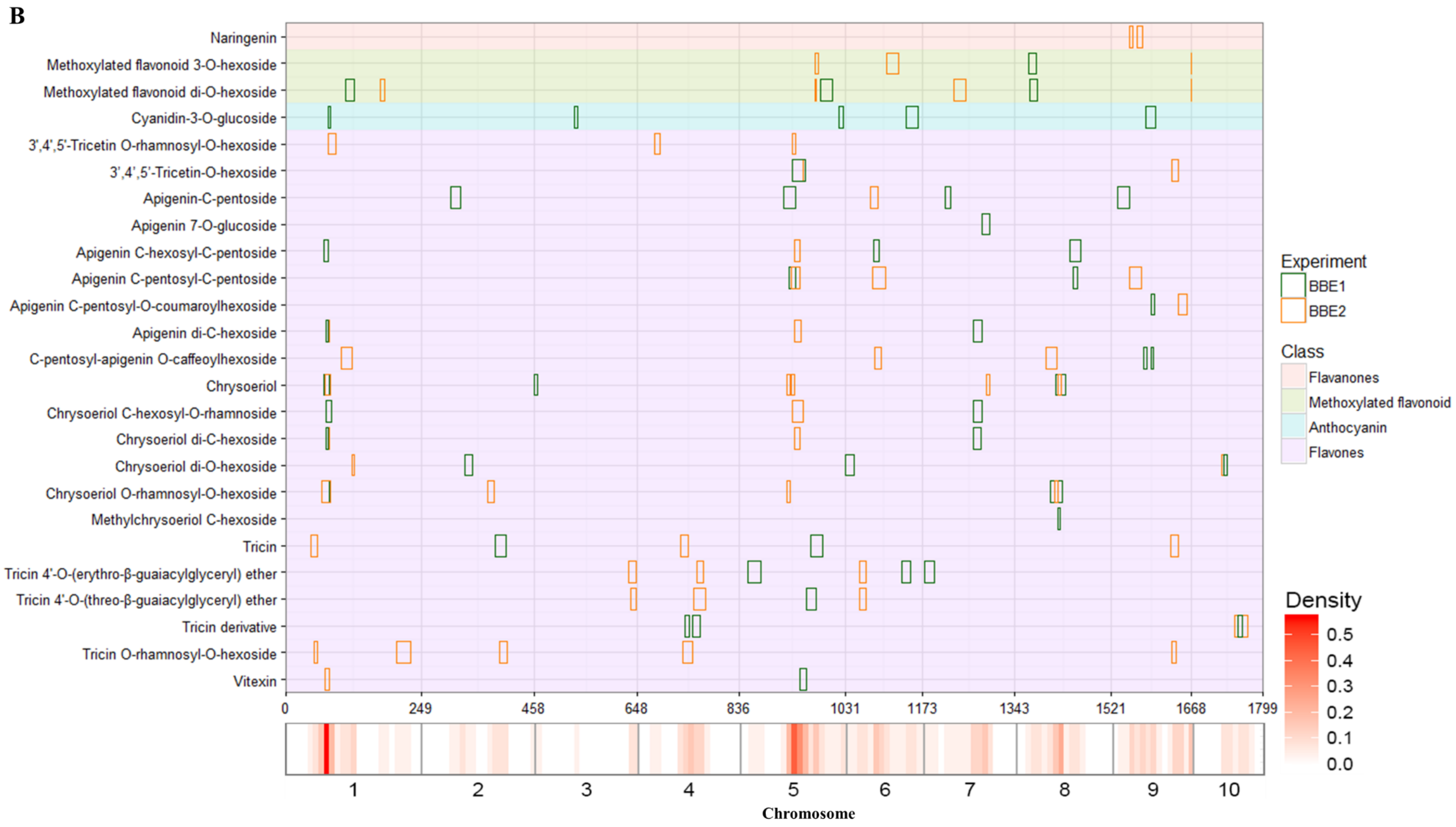
B
Naringenin
Methoxylated flavonoid 3-O-hexoside
Methoxylated flavonoid di-O-hexoside
Cyanidin-3-O-glucoside
3',4',5'-Tricetin O-rhamnosyl-O-hexoside
3',4',5'-Tricetin-O-hexoside
Apigenin-C-pentoside
Apigenin 7-O-glucoside
Apigenin C-hexosyl-C-pentoside
Apigenin C-pentosyl-C-pentoside
Apigenin C-pentosyl-O-coumaroylhexoside
Apigenin di-C-hexoside
C-pentosyl-apigenin O-caffeoylhexoside
Chrysoeriol
Chrysoeriol C-hexosyl-O-rhamnoside
Chrysoeriol di-C-hexoside
Chrysoeriol di-O-hexoside
Chrysoeriol O-rhamnosyl-O-hexoside
Methylchrysoeriol C-hexoside
Tricin
Tricin 4'-O-(erythro-β-guaiacylglyceryl) ether
Tricin 4'-O-(threo-β-guaiacylglyceryl) ether
Tricin derivative
Tricin O-rhamnosyl-O-hexoside
Vitexin
0
249
458
648
836
1031
1173
1343
1521
1668
1799
Experiment
BBE1
BBE2
Class
Flavanones
Methoxylated flavonoid
Anthocyanin
Flavones
Density
0.5
0.4
0.3
0.2
0.1
0.0
1
2
3
4
5
6
7
8
9
10
Chromosome

C

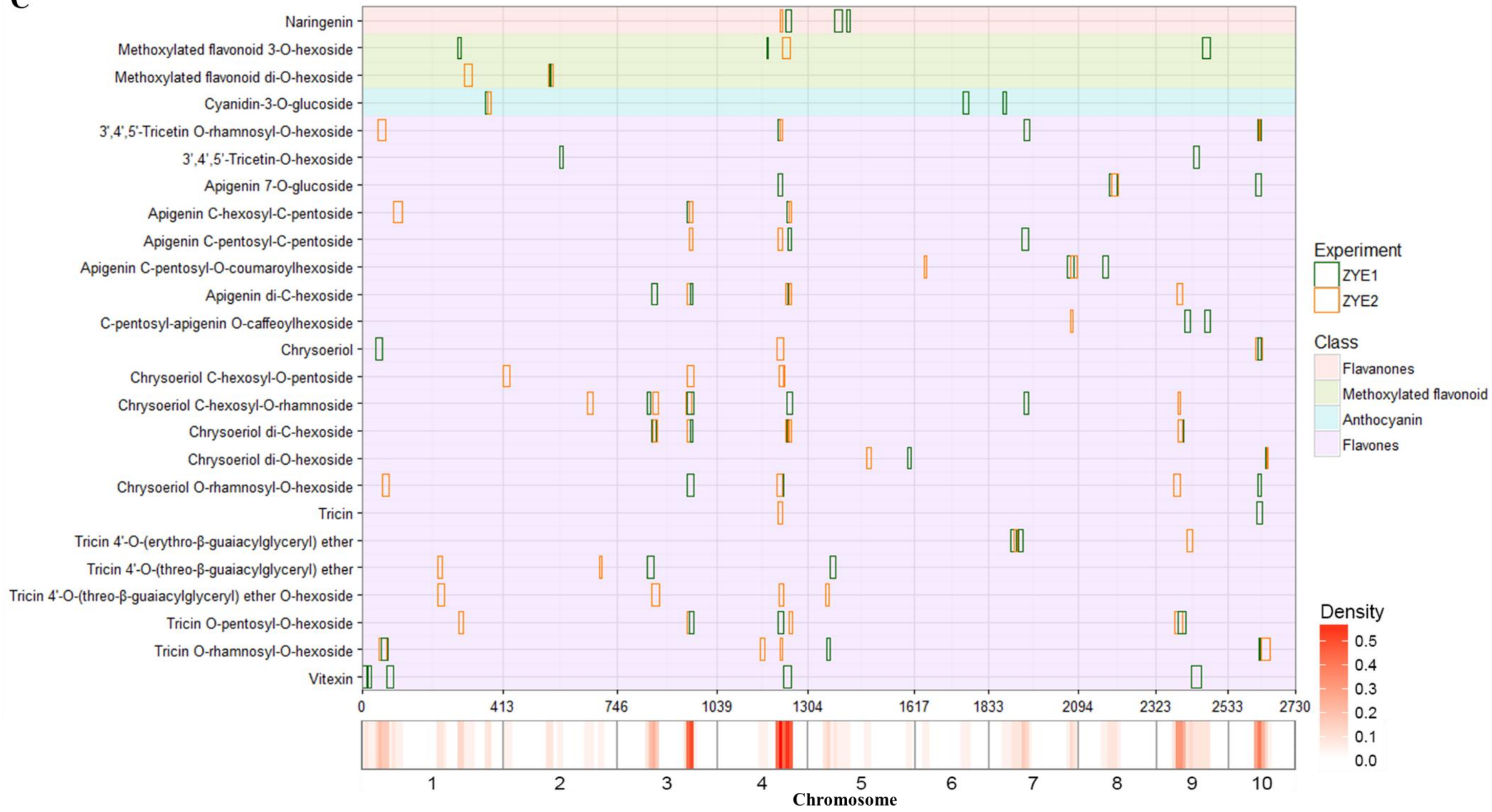

Naringenin
Methoxylated flavonoid 3-O-hexoside
Methoxylated flavonoid di-O-hexoside
Cyanidin-3-O-glucoside
3',4',5'-Tricetin O-rhamnosyl-O-hexoside
3',4',5'-Tricetin-O-hexoside
Apigenin 7-O-glucoside
Apigenin C-hexosyl-C-pentoside
Apigenin C-pentosyl-C-pentoside
Apigenin C-pentosyl-O-coumaroylhexoside
Apigenin di-C-hexoside
C-pentosyl-apigenin O-caffeoylhexoside
Chrysoeriol
Chrysoeriol C-hexosyl-O-pentoside
Chrysoeriol C-hexosyl-O-rhamnoside
Chrysoeriol di-C-hexoside
Chrysoeriol di-O-hexoside
Chrysoeriol O-rhamnosyl-O-hexoside
Tricin
Tricin 4'-O-(erythro-β-guaiacylglyceryl) ether
Tricin 4'-O-(threo-β-guaiacylglyceryl) ether
Tricin 4'-O-(threo-β-guaiacylglyceryl) ether O-hexoside
Tricin O-pentosyl-O-hexoside
Tricin O-rhamnosyl-O-hexoside
Vitexin
0
413
746
1039
1304
1617
1833
2094
2323
2533
2730
Experiment
ZYE1
ZYE2
Class
Flavanones
Methoxylated flavonoid
Anthocyanin
Flavones
Density
0.5
0.4
0.3
0.2
0.1
0.0
1
2
3
4
5
6
7
8
9
10
Chromosome

Figure S2. Chromosomal distribution of flavonoid QTLs identified in this study. (A) Chromosomal Distribution of Flavonoid QTLs Identified in AMP. QTL position and significance (represented by circle size) across the maize genome responsible for flavonoid level from E1 and E2 experiment for AMP are shown as black (AMPE1) and red (AMPE2) circle, respectively. The x axis indicates the physical positions across the maize genome in Mb. (B) and (C) Chromosomal Distribution of Flavonoid QTLs Identified in both BB and ZY populations. QTL regions (represented by the confidence interval) across the maize genome responsible for flavonoid level from E1 and E2 experiment for each RIL populations are shown as green (BBE1 and ZYE1) and orange (BBE2 and ZYE2) boxes, respectively. The x axis indicates the genetic positions across the maize genome in cM. Heat map under the x axis illustrates the density of flavonoid QTL across the genome. The window size is 10 cM for BB and ZY population and 10 Mb for AMP, respectively. Detailed information of all detected QTLs is shown in **Supplemental Tables 3 and 5**. Flavonoids from different class are marked by distinct colors as shown on the right.
