# Supplementary figures and images for: Integrated genomics-based mapping reveals the genetics underlying maize flavonoid biosynthesis
Source: BMC Plant Biol. 2017 Jan 18;17:17. doi: 10.1186/s12870-017-0972-z (PMC5242060; doi:10.1186/s12870-017-0972-z)

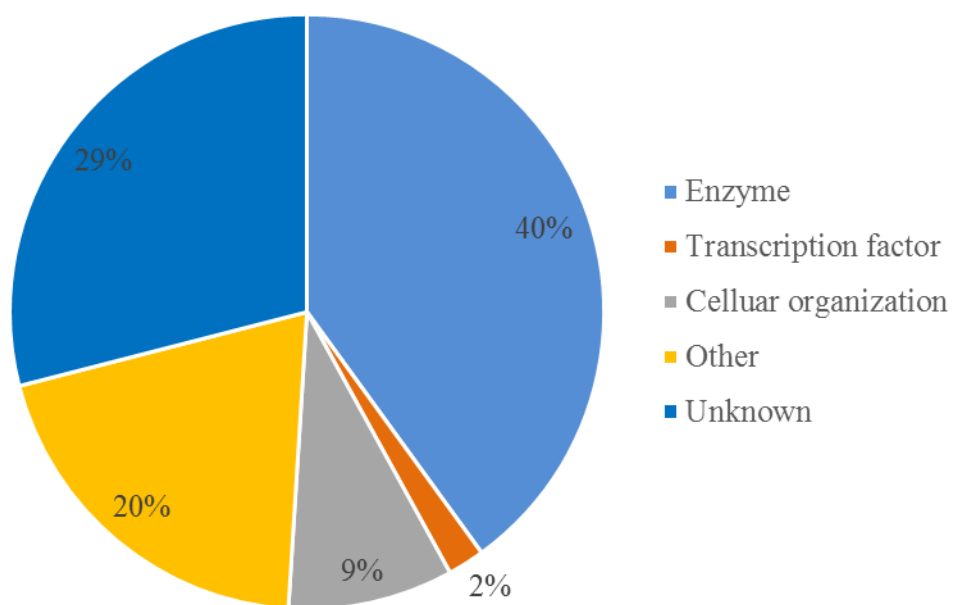

**Figure S3. Classification of candidate genes according to their functional annotations.**

Supplement: Additional file 10: Figure S3. — Classification of candidate genes according to their functional annotations. (PDF 32 kb) [file 12870_2017_972_MOESM10_ESM.pdf]

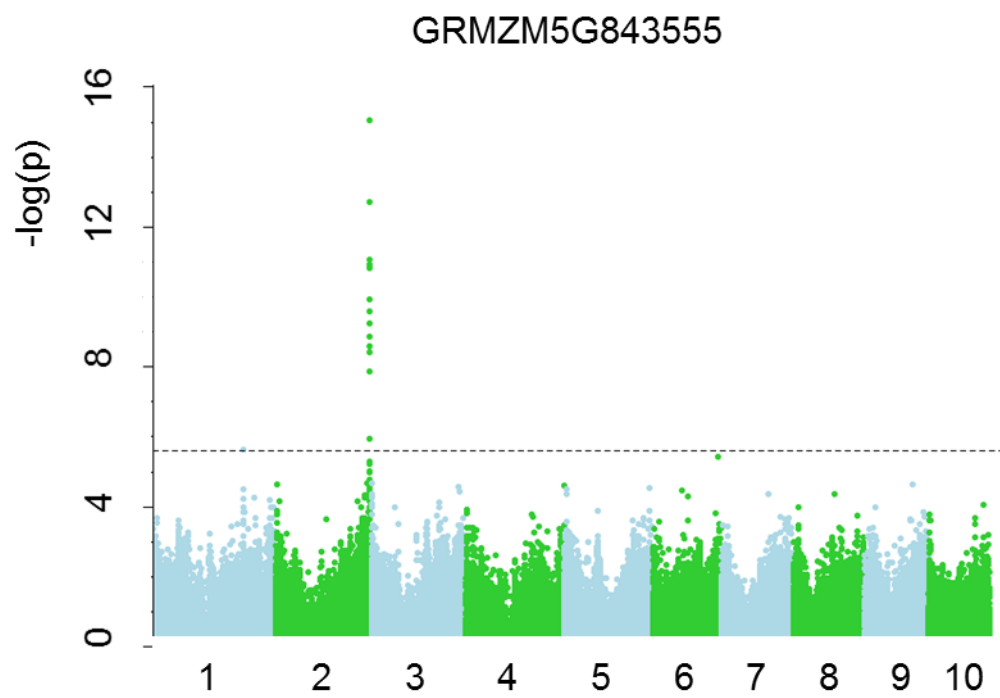

**Figure S8.** Manhattan plot showing the GWAS result of the expression level of *OXY* (GRMZM5G843555).

Supplement: Additional file 16: Figure S8. — Manhattan plot showing the GWAS result of the expression level of OXY (GRMZM5G843555). (PDF 59 kb) [file 12870_2017_972_MOESM16_ESM.pdf]
