# Supplementary material for: Integrated genomics-based mapping reveals the genetics underlying maize flavonoid biosynthesis
Source: BMC Plant Biol. 2017 Jan 18;17:17. doi: 10.1186/s12870-017-0972-z (PMC5242060; doi:10.1186/s12870-017-0972-z)
Supplement: Additional file 11: Figure S4. — Boxplots showing the distribution of flavonoids level. (PDF 158 kb) [file 12870_2017_972_MOESM11_ESM.pdf]

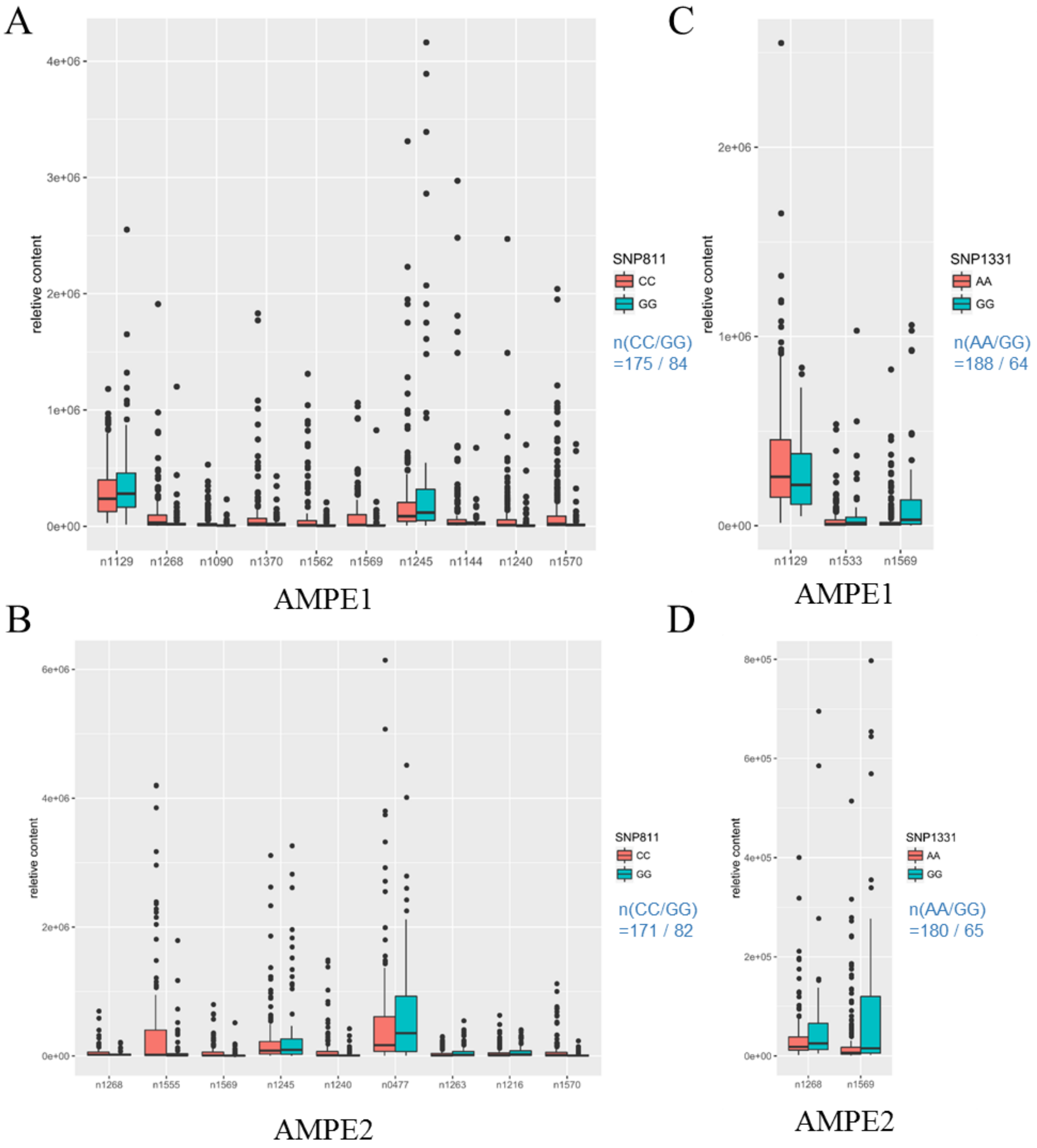

Figure S4. Boxplots showing the distribution of flavonoids level. (A-B) Boxplot for flavonoids significantly effected ($p<0.05$) by SNP811 in AMPE1 and AMPE2. (C-D) Boxplot for flavonoids significantly effected ($p<0.05$) by SNP1331 in AMPE1 and AMPE2.
