# Supplementary material for: Integrated genomics-based mapping reveals the genetics underlying maize flavonoid biosynthesis
Source: BMC Plant Biol. 2017 Jan 18;17:17. doi: 10.1186/s12870-017-0972-z (PMC5242060; doi:10.1186/s12870-017-0972-z)
Supplement: Additional file 14: Figure S6. — Bar plot of the relative flavonoid levels (fold change relative to the mean level of each flavonoid) that are significantly different between the wild type (WT) and OXY over-expression lines. (PDF 131 kb) [file 12870_2017_972_MOESM14_ESM.pdf]

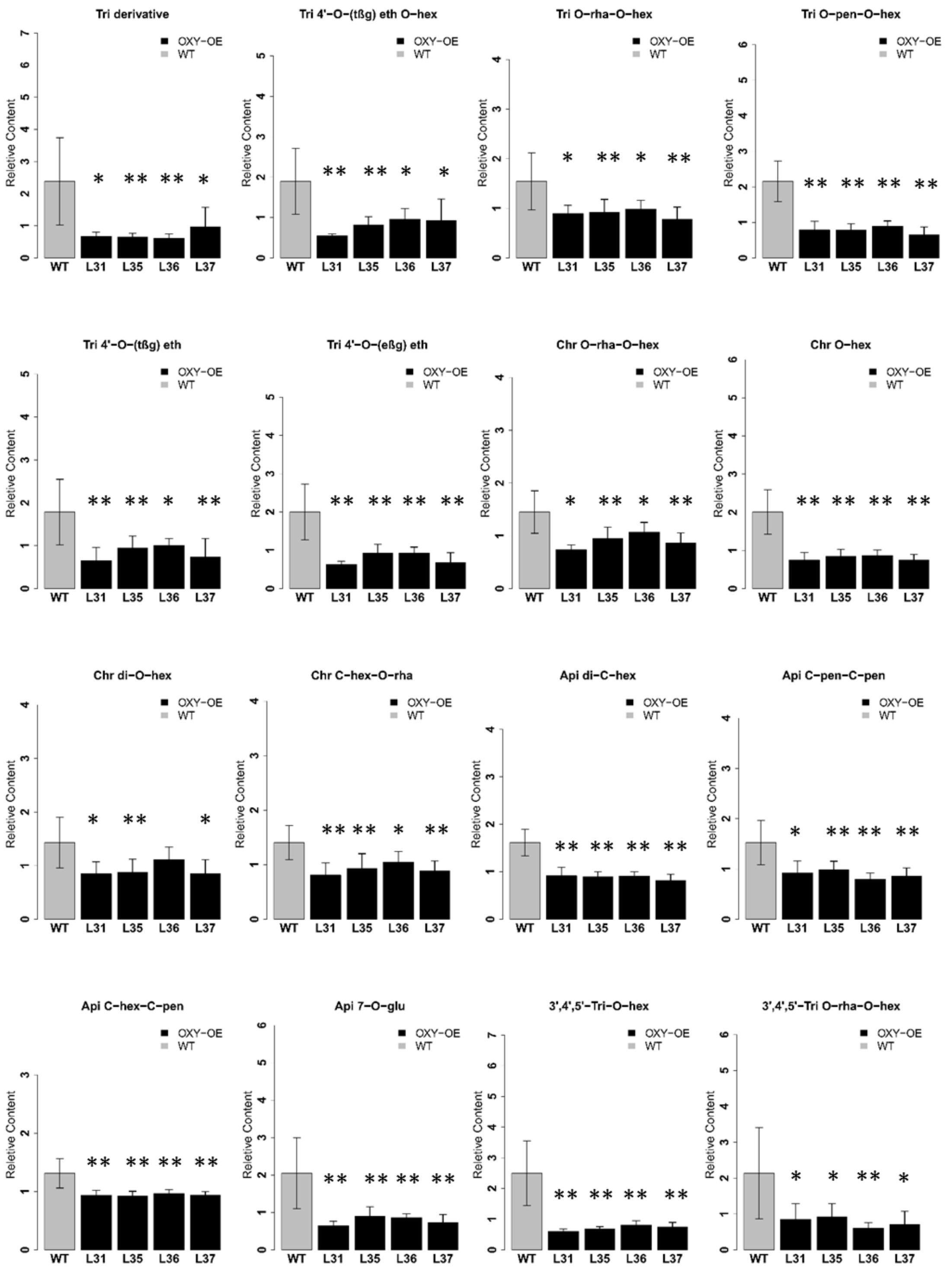

Figure S6. Bar plot of the flavonoid levels that are significantly different between the wild type (WT) and OXY over-expression lines. (n=6, 10, 7, 8 respectively), * and ** represent the significant level at 0.05 and 0.01, respectively.
