# Supplementary material for: Integrated genomics-based mapping reveals the genetics underlying maize flavonoid biosynthesis
Source: BMC Plant Biol. 2017 Jan 18;17:17. doi: 10.1186/s12870-017-0972-z (PMC5242060; doi:10.1186/s12870-017-0972-z)
Supplement: Additional file 15: Figure S7. — The sequence polymorphisms between B73 and By804 of the promoter region of gene OXY. (PDF 215 kb) [file 12870_2017_972_MOESM15_ESM.pdf]

```
zong3_P       -------GCTTGTACTACGTCAGCATCTATGCCGGCGTCAGAGCAGACACAGCTGCAG--
Yu87-1_P      AGCTTGTACTTGAGCAACGTCAGCATCTATGCCGGCGTCAAATCAAATCTTACAGACAAG
                      ****  * ************************ * ** *     * *

zong3_P       ---------------TGAATTAGCACCGGCGGTACTGAACTGGAGGAAGGAAAGGTAGAG
Yu87-1_P      CCAGACACAGCTGCAGTGTTTAGCACCGGCGG-----TACTGAACTGGAGGAAGGTAGAG
                                 *************      **** *     * *********

zong3_P       TGAATGAAGTACACTCTTGTATATACGGGAAGATTTAAGGCCTATTTGTTTCGCTAATTA
Yu87-1_P      TGAATGAAGTACACTCTTGTATATGCGGGAAGATTTAAAGCATGATTCGTTGAC----TA
              ************************ ************* ** *  **  **  *    **

              MBSII: MYB binding site involved in flavonoid biosynthetic genes regulation(Petunia hybrida)
zong3_P       ATTTGTCATACTTTATCTAACTTTTCTGACTAAAATTAGCATTTCAATTCAAACGACCAA
Yu87-1_P      ACTTGTCACACTTTGCCTAACTTTTCTATCTAAATTTAGTTATTCAGTTTGAACGACTAC
              * ****** *****  ***********  ***** ****   **** **  ****** *

zong3_P       CCTTAAAT-AAAGTGTGATATAGTTAGC----------CATAAACCAAACATGTCAAGAA
Yu87-1_P      TCTTAAGCAAAAGTGTGGTATAGTTAGTTATAAATCAAACAGACCTTTATTCTCCAATAA
               *****   ******** *********               * *   *     *** **

zong3_P       CCTTTTATAGTGAACCATTGGACTATTGTTCATCTCCTACGTGCGGTTGGGGAAAGTTCG
Yu87-1_P      CCTTTTATAGTGAACCAATGGACTATTGTTCATCTCCTACGTGCGGTTAGGGAAAGTTCG
              ***************** ****************************** ***********
```

Figure S7. The sequence polymorphisms between B73 and By804 of the promoter region of gene *OXY*. The coloured region indicates the Myb binding site involved in flavonoids biosynthetic genes regulation. The yellow and blue shows the different alleles occurs in the MBSII binding site.
